# Transcriptional Profile of *Mycobacterium tuberculosis* in an *in vitro* Model of Intraocular Tuberculosis

**DOI:** 10.3389/fcimb.2018.00330

**Published:** 2018-10-02

**Authors:** Sudhanshu Abhishek, Uma Nahar Saikia, Amod Gupta, Reema Bansal, Vishali Gupta, Nirbhai Singh, Suman Laal, Indu Verma

**Affiliations:** ^1^Department of Biochemistry, Postgraduate Institute of Medical Education and Research, Chandigarh, India; ^2^Department of Histopathology, Postgraduate Institute of Medical Education and Research, Chandigarh, India; ^3^Department of Ophthalmology, Postgraduate Institute of Medical Education and Research, Chandigarh, India; ^4^Department of Pathology, New York University Langone Medical Center, New York, NY, United States; ^5^Veterans Affairs New York Harbor Healthcare System, New York, NY, United States

**Keywords:** *Mycobacterium tuberculosis*, RPE, intraocular tuberculosis, transcriptome, electron microscopy, intracellular adaptation, pathogenesis, vitreous samples

## Abstract

**Background:** Intraocular tuberculosis (IOTB), an extrapulmonary manifestation of tuberculosis of the eye, has unique and varied clinical presentations with poorly understood pathogenesis. As it is a significant cause of inflammation and visual morbidity, particularly in TB endemic countries, it is essential to study the pathogenesis of IOTB. Clinical and histopathologic studies suggest the presence of *Mycobacterium tuberculosis* in retinal pigment epithelium (RPE) cells.

**Methods:** A human retinal pigment epithelium (ARPE-19) cell line was infected with a virulent strain of *M. tuberculosis* (H37Rv). Electron microscopy and colony forming units (CFU) assay were performed to monitor the *M. tuberculosis* adherence, invasion, and intracellular replication, whereas confocal microscopy was done to study its intracellular fate in the RPE cells. To understand the pathogenesis, the transcriptional profile of *M. tuberculosis* in ARPE-19 cells was studied by whole genome microarray. Three upregulated *M. tuberculosis* transcripts were also examined in human IOTB vitreous samples.

**Results:** Scanning electron micrographs of the infected ARPE-19 cells indicated adherence of bacilli, which were further observed to be internalized as monitored by transmission electron microscopy. The CFU assay showed that 22.7 and 8.4% of the initial inoculum of bacilli adhered and invaded the ARPE-19 cells, respectively, with an increase in fold CFU from 1 dpi (0.84) to 5dpi (6.58). The intracellular bacilli were co-localized with lysosomal-associated membrane protein-1 (LAMP-1) and LAMP-2 in ARPE-19 cells. The transcriptome study of intracellular bacilli showed that most of the upregulated transcripts correspond to the genes encoding the proteins involved in the processes such as adherence (e.g., *Rv1759c* and *Rv1026*), invasion (e.g., *Rv1971* and *Rv0169*), virulence (e.g., *Rv2844* and *Rv0775*), and intracellular survival (e.g., *Rv1884c* and *Rv2450c)* as well as regulators of various metabolic pathways. Two of the upregulated transcripts (*Rv1971, Rv1230c*) were also present in the vitreous samples of the IOTB patients.

**Conclusions:** *M. tuberculosis* is phagocytosed by RPE cells and utilizes these cells for intracellular multiplication with the involvement of late endosomal/lysosomal compartments and alters its transcriptional profile plausibly for its intracellular adaptation and survival. The findings of the present study could be important to understanding the molecular pathogenesis of IOTB with a potential role in the development of diagnostics and therapeutics for IOTB.

## Introduction

Intraocular tuberculosis (IOTB) is a unique extrapulmonary manifestation (Gupta et al., 2016) of the disease in TB endemic as well as non-endemic countries (Patel et al., 2013; Lee et al., 2016). No prospective data are available on the population-based prevalence of IOTB either from the low-endemic or the high-endemic TB regions in the world (Gupta et al., 2016). However, among the uveitis clinic population, the prevalence of TB as an etiology of uveitis has varied from 0.5% in the USA, 4% in the People's Republic of China, 6.31% in Italy, 6.9% in Japan, 9.86% in north India, 10.5% in Saudi Arabia, and 11.4% in Iraq where TB is endemic (Shakarchi, 2015). Altogether, the prevalence ranges from 1–4% (non-endemic) to 10–26% (endemic) in different areas of the world (Lee et al., 2016). A recent report from India showed that 5.2% of patients with uveitis also had sputum positive pulmonary or extrapulmonary TB (Gogia et al., 2018). Another retrospective multinational cohort study including 801 patients from 25 ophthalmology referral centers demonstrated good treatment response in TB uveitis patients (Agrawal et al., 2017). More importantly, IOTB is becoming a major health problem, similar to other types of extrapulmonary TB (Dalvin and Smith, 2017), not only due to the increasing prevalence but also due to the identification of drug-resistant cases (Sharma et al., 2018).

Intraocular tuberculosis is a significant cause of inflammation and visual morbidity, particularly in TB endemic countries (Gupta et al., 2015; Shakarchi, 2015). It poses clinical, diagnostic, and therapeutic challenges due to the protean clinical presentations of IOTB within a single organ (Gupta et al., 2015). Unavailability of adequate human samples from the eye, an immune-privileged site, and failure in detecting the presence of live bacteria further makes it difficult to understand the pathogenesis of IOTB (Shechter et al., 2013; Gupta et al., 2015; Lee et al., 2016). In view of the increasing number of cases of IOTB along with the diagnostic dilemma associated with this disease, it is very important to study the molecular pathogenesis of IOTB using the relevant experimental models. Studies using experimental TB models, such as animal and cell lines, have already been proven useful for understanding the various aspects of pulmonary as well as extrapulmonary forms of TB (Talaat et al., 2004; Jain et al., 2006; Be et al., 2008; Doycheva et al., 2010; Krishnan et al., 2010; Scordo et al., 2016; Fonseca et al., 2017; Zhan et al., 2017). *Mycobacterium tuberculosis* primarily localizes in the lung and is taken up by the alveolar macrophages which are also involved in the transport of bacilli by the hematogenous route (Henderson et al., 1963; Balasubramanian et al., 1994; Danelishvili et al., 2003) to various other organs where it remains dormant until it gets activated and produces extrapulmonary TB disease (Tufariello et al., 2003; Barrios-Payán et al., 2012). So far it is not known how and where on reaching the eye, *M. tuberculosis* is localized and activates sight-threatening inflammation/uveitis. Although recent clinical reports highlight that *M. tuberculosis* can affect any tissue of the eye, primarily the posterior part of the eye is involved due to high oxygen tension (Dalvin and Smith, 2017; Moharana et al., 2018). The late-stage IOTB has been found to occur in retina as retinitis and retinal vasculitis (Doycheva et al., 2010; Gupta et al., 2015), and in a clinical sample representing granulomatous uveitis, acid-fast bacilli (AFB) have been shown to be present in the retinal pigment epithelium (RPE) cells (Rao et al., 2006). Thus, the RPE cells—the non-professional phagocytic cells in the eye—have been considered as a probable host for the survival and replication of *M. tuberculosis* (Gupta et al., 2007), and reactivation of these sequestered organisms may lead to the recurrence of IOTB (Patel et al., 2013). Studies on the intracellular *M. tuberculosis* in both alveolar macrophages (professional) and alveolar epithelial (non-professional) cells have indicated that (Danelishvili et al., 2003) soon after invasion, *M. tuberculosis* gets localized in a cytoplasmic compartment known as phagosomes, and acquires the fusion with late endosomal/lysosomal markers but inhibits the biogenesis of phagolysosomes for its intracellular survival (Hasan et al., 1997; Huynh et al., 2007).

Recently, a human fetal retinal pigment epithelial cell line model has been reported to phagocytize an avirulent form of mycobacteria, H37Ra, with the involvement of host toll-like receptors (Nazari et al., 2014). However, a cell line model for IOTB using virulent *M. tuberculosis* (H37Rv) for elucidating the key determinants (genes/proteins) of disease pathogenesis is still lacking. Identification of transcriptional signatures of the bacteria is key to understanding how the bacteria colonize, invade, or replicate in host microenvironment (Talaat et al., 2004) and has been proven to be a valid approach in understanding the pathogenesis of TB (Ward et al., 2010). Transcriptional changes in pathogen (*M. tuberculosis*) have been studied in well-defined *in vitro* culture systems (Lin et al., 2016) as they efficiently mimic the host environment and this has improved our knowledge on the host-pathogen interactions (Nandy et al., 2018). These studies have led to the identification of transcripts and pathways specific to growth conditions involved in the survival of the pathogen in the unfavorable lysosomal microenvironment (Lin et al., 2016). Thus, transcriptomic knowledge is possibly valuable for the design of new drugs, vaccines, and new approaches for controlling TB (Aguilar-Ayala et al., 2017). At present, various critical key gaps exist in the field of IOTB as it is still not known how the mycobacteria alter its transcriptional profile to support its survival and replication in the ocular environment.

Thus, the current study is an attempt to investigate some of these aspects. We have developed a well-defined *in vitro* RPE cell line model of IOTB and utilized it to examine the intracellular fate of *M. tuberculosis* using late endosomal/lysosomal cell surface markers and *M. tuberculosis* transcriptome to identify the key determinants involved in the pathogenesis of IOTB.

## Materials and methods

### Cell line and *M. tuberculosis* strain

All the experiments were carried out in accordance with the rules and regulations set forth by the Institute's Ethics Committee (IEC) and Institute's Biosafety Committee (IBC), Post Graduate Institute of Medical Education and Research (PGIMER), Chandigarh. Human samples were obtained from the ocular samples repository for ophthalmology research (#NK/3323/study/186).

*M. tuberculosis* H37Rv was grown in Sauton's media (0.5 g of potassium dihydrogen phosphate, 0.5 g of magnesium sulfate, 2 g of citric acid, 0.05 g of ferric ammonium citrate, and 4 g of L-asparagine in 900 mL of double distilled water. Once dissolved, 60 mL of glycerol and 0.05% Tween-80 were added and pH of the medium was adjusted to 7.2–7.4 with sodium hydroxide solution).

Adult retinal pigment epithelium-19 (ARPE-19) (ATCC® CRL-2302™) cell line was maintained in Dulbecco's Modified Eagle Medium Nutrient Mixture F-12 (DMEM/F-12) media (Thermo Scientific #12400024) supplemented with sodium bicarbonate (1.2 g/L), penicillin (100 U/ml), streptomycin (100 μg/ml), 10% FBS, and 5% carbon dioxide (CO_2_) at 37°C. The ARPE-19 cells were resuspended in fresh growth media without antibiotics prior to *M. tuberculosis* infection.

### *In vitro* model of intraocular tuberculosis

#### H37RV inoculum

Single cell suspension of H37Rv was prepared using log phase H37Rv culture grown in Sauton's media with minor modifications (Napier et al., 2011). The H37Rv culture was centrifuged at 3,000 rpm for 5 min and the supernatant was removed. The pellet was resuspended and washed twice using phosphate buffer saline supplemented with 0.05% Tween-80 buffer (PBS-T; pH-7.2). The washed pellet thus obtained was suspended in 20 ml of PBS-T buffer with 3 mm glass beads (Merck), vortexed for 15 s to disperse clumps, and the suspension was kept standing for 10 min at room temperature. Ten milliliters of this suspension was collected in fresh 50 ml falcon tubes, mixed with fresh 3 mm of glass beads, vortexed again for 15 s, followed by standing for 10 min at room temperature. This step was repeated three times and the single cell suspension of H37Rv thus obtained was matched with MacFarland standards, and the appropriate dilutions were prepared in fresh antibiotic-free DMEM/F-12 medium for infecting the ARPE-19 cells.

#### Adherence, invasion, and multiplication of *M. tuberculosis* H37RV in ARPE-19 cells

The standard methodology for *in vitro* infection of epithelial cells with H37Rv was followed with minor modifications (Mehta et al., 1996; Napier et al., 2011). The ARPE-19 monolayer was washed (three times) with antibiotic-free DMEM/F-12 medium followed by addition of fresh antibiotic-free DMEM/F-12 medium supplemented with 2% FBS. The cells were infected with a single cell suspension of H37Rv in the same medium at a multiplicity of infection (MOI) of 10 bacteria per cell (10:1) for 2-h (adherence time-point). The adherence of *M. tuberculosis* to ARPE-19 cell line was observed through scanning electron microscopy (SEM) and quantified by colony forming units (CFU) assay (Mehta et al., 1996; Ramsugit and Pillay, 2016). Two-hour post infection, the monolayers of the infected and uninfected cells were washed three times with warm antibiotic-free DMEM/F-12 medium and replaced by fresh antibiotic-free DMEM/F-12 medium supplemented with 2% FBS and amikacin (20 μg/mL) to kill the extracellular bacilli at 37°C under 5% CO_2_ for 2-h (0-day post infection; dpi). At this time-point, the invasion of bacilli in the ARPE-19 cells was monitored by transmission electron microscopy (TEM) and quantitatively confirmed by CFU enumeration. Following 2 h of amikacin treatment, all the cultured cells (infected and uninfected) were maintained in fresh antibiotic-free DMEM/F-12 medium supplemented with 1% FBS and amikacin (2 μg/mL) (to preclude any extracellular growth of *M. tuberculosis*) to monitor the intracellular replication at 1−, 3−, and 5-dpi by the CFU assay. Both the infected and uninfected ARPE-19 cells were incubated at 37°C under 5% CO_2_ at all the time-points.

#### Scanning electron microscopic examination

The ARPE-19 cell line was cultured at a cell density of 2 × 10^5^ cells/well on 8 mm coverslips in 24-well tissue culture plates. At 15- and 30-min of infection, both the infected and uninfected ARPE-19 cells on the coverslips were fixed using a fixative solution [glutaraldehyde 2.5% (v/v), paraformaldehyde fixative 2% (w/v), phosphate buffer 0.1 M] for 24 h. After fixation, the coverslips were washed with PBS, dehydrated using ethanol, air dried, platinum coated, and observed under JSM-IT300LV scanning electron microscope (JEOL, Tokyo Japan) at Central Sophisticated Instruments Cell (CSIC), PGIMER, Chandigarh, India. The H37Rv inoculum used for infection was also fixed on coverslips and processed for SEM imaging as a positive control.

#### Transmission electron microscopic examination

To visualize the internalized bacilli within the RPE cells, TEM studies were conducted. The ARPE-19 cells were seeded at a density of 1 × 10^6^ cells in T75 tissue culture flasks. At 0-, 1-, and 3-dpi, the infected and uninfected ARPE-19 cells were washed, de-adhered, and the cell pellets were collected by centrifugation at 2,000 rpm for 10 min. The cell pellets were then fixed in fixative solution in 0.2 M Sorensen's phosphate buffer (sodium dihydrogen phosphate 0.2 M, sodium hydrogen phosphate 0.2 M, sucrose 7.5%, and glutaraldehyde 4%) for 2–4 h followed by a mild spin and fixation in 1% osmium tetroxide and dehydration in ethanol series. The cells were then infiltrated with EPON mixture and propylene oxide (1:1) for 2 h at room temperature and then embedded in EPON resin, followed by polymerization at 60°C for 24 h. Sections of 0.5 μm thickness were cut using an ultramicrotome (Reichert-Jung, Leica, Germany) and stained with 0.5% toluidine blue to confirm the presence of cells. Further, 60 nm ultra-thin sections were cut and mounted on nickel grids (300 mesh). The sections were double-stained with uranyl acetate and lead citrate and then examined by TEM at the Department of Histopathology, PGIMER, Chandigarh, India and photographed. The H37Rv inoculum used for infection during the study was also fixed and processed similarly for TEM analysis.

#### Colony forming units enumeration

The ARPE-19 cell line was cultured at a cell density of 2 × 10^5^ cells/well in 24-well tissue culture plates. The infected and uninfected ARPE-19 cells at the indicated time points were lysed by treatment with 0.5 ml of 0.1% Triton X-100 for 5 min, and serial dilutions of the infected and non-infected cell lysates (1:10, 1:100, and 1:1,000) prepared in 7H9 broth were plated on 7H11 agar supplemented with 10% OADC. H37Rv used during infection (inoculum) was also plated at similar dilutions. Colonies were counted after 3–4 weeks of incubation at 37°C/5% CO_2_. The colonies of the respective time points at each dilution were counted and averaged (from two independent experiments with two independent wells at each time-point) and the percent adherence and invasion were calculated with respect to the initial inoculums as explained in the legend of Table 2. Intracellular multiplication was calculated in terms of fold CFU (mean CFU at each time-point divided by the day 0 CFU) (Scharn et al., 2016).

#### Cell viability assay

The effect of *M. tuberculosis* infection (MOI 10:1) on the viability of ARPE-19 cells at different time-points was studied by MTT (3-[4,5-methylthiazol-2-yl]-2,5-diphenyl-tetrazolium bromide) assay. The RPE cells (10^4^ cells/100 μL/well) were plated in a 96-well tissue culture plate. The uninfected and infected ARPE-19 cells (at 0-, 1-, 3-, and 5-dpi) were washed and incubated with 100 μL of fresh DMEM/F-12 medium containing 10 μL of MTT solution (5 mg/mL) at 37°C for 4 h. After incubation, 100 μL of a freshly prepared solution containing 0.01 M HCl and 10% SDS was added to the wells to dissolve formazan crystals. Following overnight incubation, the absorbance was measured on an ELISA plate reader at 570 nm, and the viability of the uninfected cells at the respective time-points was considered as 100%. At each time-point, the wells containing DMEM/F-12 medium, MTT and SDS/HCl solution without ARPE-19 cells were used as a blank. The data presented are the mean ± SEM of O.D. at A570 (uninfected and infected wells), and percent cytolysis was calculated by dividing the mean A570 of the test wells by mean A570 of the control cells × 100.

### Intracellular fate of *M. tuberculosis* in the retinal pigment epithelial cells

To understand the intracellular fate of *M. tuberculosis* inside the RPE cells, colocalization of *M. tuberculosis* and late endosomal/lysosomal markers (LAMP-1, lysosomal-associated membrane protein-1 and LAMP-2, lysosomal-associated membrane protein-2) were studied during the course of infection in the ARPE-19 cells (Hasan et al., 1997; Huynh et al., 2007).

#### Labeling of *M. tuberculosis*

The H37Rv was labeled before infection with PKH26 dye using MINI 26 Sigma kit which stably incorporates a yellow-orange fluorescent dye (PKH26) into the lipid regions of the cell membrane. A single cell suspension containing 2 × 10^7^ bacilli was washed and suspended in a 2X cell suspension solution (1 mL of diluent C and 1 mL 2X dye solution). The mix was incubated for 1–5 min with periodic mixing at ambient temperature (20–25°C) in the dark. After the completion of labeling, the bacterial pellets were washed twice with PBS and resuspended in antibiotic-free DMEM/F-12 medium for infection of ARPE-19 cells.

#### Confocal microscopy

The ARPE-19 cells were cultured on 14 mm glass coverslips in a 24-well tissue culture plate at a cell density of 2 × 10^5^ cells and were infected with PKH26-labeled H37Rv at MOI (10:1) as explained above. Post 2 h of amikacin (20 μg/ml) treatment, the infected and uninfected monolayer cells were washed three times with warm antibiotic-free DMEM/F-12 medium and the coverslips were collected to study the intracellular bacilli at different time points (0-, 1-, and 3-dpi). The ARPE-19 monolayer cells on the coverslips were fixed with 4% paraformaldehyde for 15 min, permeabilized with 0.05% saponin in PBS for 5 min, and blocked with 5% serum in PBS for 2 h at room temperature or overnight at 4°C. The cells were further incubated with primary antibodies for endosomal/lysosomal markers, i.e., anti-human -LAMP-1 and -LAMP-2 at a concentration of 1 and 2 μg/ml, respectively for, 2 h followed by washing thrice with PBS and incubation with FITC labeled anti-rabbit IgG secondary antibody. The ARPE-19 cells were washed again 3 times and 4′6′-diamidino-2-phenylindole dihydrochloride (DAPI) was added to the cells at a concentration of 2 ng/ml and incubated for 1 min to stain the nuclei. The coverslips were mounted in glycerol: saline (v/v, 1:1) on glass slides and the images were acquired with 100X oil immersion objective and standard filters using a confocal microscope (FLUOVIEW FV1000). Further images were captured and processed using FV10-ASW 3.0 software (Olympus) followed by colocalization analysis using Coloc2-ImageJ plugin. All the steps were performed at ambient temperature (20–25°C). Three coverslips at each time-point were analyzed each for infected and uninfected cells.

### Transcriptome profiling of *M. tuberculosis* in *in vitro* model of intraocular tuberculosis

The ARPE-19 monolayers were infected with *M. tuberculosis* at MOI of 10:1 as explained above and 3-dpi RNA was isolated from intracellular bacilli. A pool of five T225 flasks, each containing 2 × 10^7^ monolayer cells, was infected with H37Rv, and the total RNA was isolated as one sample experiment to study the whole *M. tuberculosis* genome through microarray. The RNA isolated from *M. tuberculosis* H37Rv inoculum used for infection was taken as the reference for comparison and calculation of the change in mycobacterial gene expression in ARPE cells. The data were obtained and analyzed from three independent experiments.

#### *M. tuberculosis* ribonucleic acid isolation

For the isolation of *M. tuberculosis* RNA from the infected ARPE-19 cells, the eukaryotic RNA was removed by treating the infected monolayer cells with GTC solution (4 M guanidine thiocyanate, 0.5% sodium-N-lauryl sarcosine, 25 mM tri-sodium citrate, 0.1 M β-2-mercaptoethanol, and 0.5% Triton-80) for 2–5 min. The lysate was centrifuged at 5,000 g for 20 min at 4°C to collect the pellet to which 1–2 mL of the TRIzol solution was added followed by addition of 0.1 mm zirconia beads for bead-beating. The TRIzol solution was collected by centrifugation at 16,000 g for 1 min at 4°C to which 1/5th volume of chloroform was added followed by incubation at RT for 10 min. After centrifugation, 250 μL of chilled isopropanol and 250 μL 0.1 M sodium acetate were added to the aqueous phase and kept at −20°C for 2 h. The samples were thereafter centrifuged at 12,000 g for 15 min at 4°C to pellet down the precipitates of RNA. Washing of RNA pellet was done using 1 mL of 75% chilled ethanol followed by centrifugation at 7,500 g for 5 min at 4°C. The pellet was air dried for 3–5 min followed by addition of nuclease-free water to the RNA pellet. RNA was quantitated and treated with Turbo DNase (2 U/μl; Ambion) as per manufacturer's instructions. The DNase-treated RNA of all the samples was purified using the Mini Elute Clean-up kit (Qiagen) and RNA amount, purity, and quality were determined by nanodrop and Agilent 2100 bioanalyzer.

#### Microarray sample preparation and array processing

Input RNA (100 ng) from experimental and control samples were amplified using T7 RNA polymerase and simultaneously labeled with Cyanine 3-CTP using One-Color Microarray-Based Low Input Quick Amp WT Labeling Protocol (Agilent Technologies). Using the RNeasy Mini kit, the labeled/amplified cRNA samples were purified and quantitated through NanoDrop ND-1000 UV-VIS Spectrophotometer. Cyanine 3 dye concentration (pmol/μL), RNA absorbance ratio (260/280 nm), and cRNA concentration (ng/μL) were recorded to determine the yield and specific activity of each reaction. The yield (μg of cRNA) was calculated using the formula, concentration of cRNA × 30 μL (elution volume)/1,000, and the specific activity (pmol Cy3 per μg cRNA) was calculated as the concentration of Cy3/concentration of cRNA × 1,000. The samples with a specific activity of more than 15 were used for hybridization to custom design the microarray slides to study the *M. tuberculosis* gene expression profile. One microgram of labeled cRNA was used from each experimental and control sample for hybridization to Agilent whole *M. tuberculosis* Genome Oligo Microarrays slides (G2509F, GE 8X15K, printed with Agilent SurePrint® technology, USA) using Agilent Microarray Hybridization Chamber Kit. After hybridization, the array slides were washed and processed for scanning on the Agilent Sure Scan Microarray Scanner using one-color scan setting for 8X15K array slides. The scanned image (.tiff) file was analyzed with Agilent Feature Extraction (FE) software (version 12.0.3.1) using default parameters. The raw data for each hybridization (experimental and control) as well as the final processed data (normalized data) have been submitted as per Minimum Information about a Microarray Experiment (MIAME) guidelines and is available at the Gene Expression Omnibus website (http://www.ncbi.nlm.nih.gov/geo/query/acc.cgi?acc=GSE115292) with accession number GSE115292.

#### Microarray data analysis

The.tiff image file was utilized to generate a quality control (QC) report for each sample (experimental and control) as per the Agilent FE protocol. For each sample, from FE software, QC metric sets, thresholds, and charting tools were determined, and the resulting text files (.txt) of the control and experimental samples were analyzed for data normalization, statistical significance, and gene expression analysis using Agilent Technologies GeneSpring® GX software (version 14.9). Significantly modulated genes were selected based on Benjamini–Hochberg procedure (Benjamini and Hochberg, 1995, 2000; Keselman et al., 2002; Pawitan et al., 2005) with a fold change >2 and *p* < 0.05, based on previous microarray studies with *M. tuberculosis* (Sharma et al., 2017).

#### Validation of *M. tuberculosis*

Transcripts *M. tuberculosis* gene-specific primers (Supplementary Table 1) were designed using NCBI Primer-BLAST tool. The microarray expression data was validated through quantitative RT-PCR on Qiagen Rotor-Gene Q real-time PCR machine using SYBR Green dye. Unamplified RNA from experimental (two independent experiments) and control (H37Rv) samples were used for cDNA synthesis (Thermo cDNA synthesis kit, #AB1453). Each sample cDNA along with the specific no enzyme control (NEC) was run in duplicate using specific cycling conditions for each gene. *M. tuberculosis* 16s gene transcript level was used as endogenous control. After completion, real-time software (Rotor-Gene Q Series Software) was used to determine the amplification plot (C_T_, cycle threshold) and to analyze the melt curve for the product specificity. Relative quantitation of target gene expression was carried out using the comparative C_T_ method (ΔΔC_T_) (Livak and Schmittgen, 2001). The C_T_ of the transcripts which showed the difference of less than 5C_T_ in comparison to its respective NEC was corrected using the formula (Laurell et al., 2012)

$$\begin{array}{l} \left\{-\text{Lo}{\text{g}}_{2}\left(2^{\wedge }-\text{RT}+\right)-\left(2^{\wedge }-\text{RT}-\right)\right\}, \end{array}$$

where,

RT+ is C_T_ of transcripts observed in cDNA sample,

RT– is C_T_ of transcripts observed in no enzyme control (NEC) sample.

Further, Graph-Pad Prism software was used for unpaired two-tailed *t-*test for statistical analysis and *p* < 0.05 was considered statistically significant.

### *Mycobacterium tuberculosis* transcripts in human intraocular tuberculosis samples

We were further interested to study if the *M. tuberculosis* transcriptional signatures identified in the *in vitro* model of IOTB are also present under *in vivo* conditions to evaluate their utility as potential molecular biomarkers in human IOTB. For this, qualitative real-time PCR was used as a tool which is considered to be advantageous over conventional PCR (Peng et al., 2016) and can also be manipulated as a diagnostic approach. Thus, three *M. tuberculosis* transcripts (*Rv1230c, Rv1971*, and *Rv3872*) observed to be upregulated in ARPE-19 cells were also examined in the human vitreous samples from two different categories (possible and confirmed IOTB). Two hundred microliters of each vitreous sample (*n* = 10; possible = 3, and confirmed = 7) and H37Rv (positive control) was utilized for total RNA isolation as explained above. The cDNA was synthesized (Thermo cDNA synthesis kit, #AB1453) from isolated RNA along with no enzyme control (NEC) reactions. The two reactions differed from each other in terms of presence (reverse transcriptase, RT+) or absence (no enzyme control NEC, RT–) of the reverse transcriptase enzyme. Additionally, no template controls were also run. All the samples along with the positive and negative controls were then analyzed for specific melt curves and C_T_ values. A C_T_ value < 35 was taken as positive amplification. The amplification was considered due to DNA (D+) if the C_T_ value in RT– sample was lesser than or equal to RT+ sample and due to RNA (R+) if the C_T_ in RT+ sample was lesser than or equal to RT– sample.

## Results

In the present study, an *in vitro* RPE cell line model of IOTB has been established to monitor the adherence, invasion, intracellular replication, and the fate of *M. tuberculosis*. Using this *in vitro* model of IOTB, the whole genome microarray was carried out to determine the *M. tuberculosis* transcriptional signatures to understand the key determinants involved in the survival of mycobacteria in ocular environment and pathogenesis of IOTB.

### *In vitro* model of intraocular tuberculosis

#### *M. tuberculosis* adheres, invades, and multiplies in the ARPE-19 cell line

**Figure 1:**
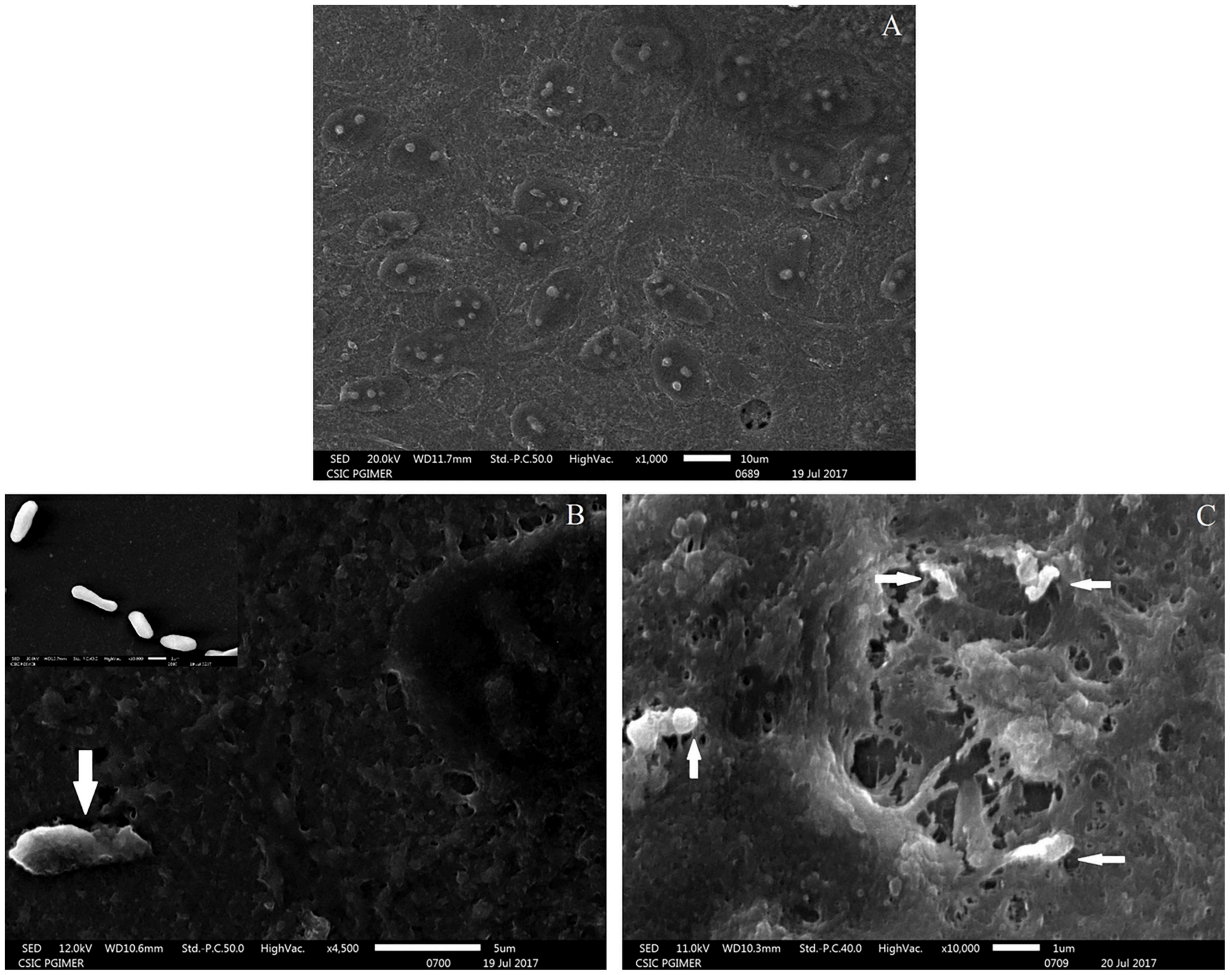
Scanning electron microscopy images of uninfected and *M. tuberculosis* infected ARPE-19 cells. **(A)** Electron micrograph of uninfected ARPE-19 monolayer cells. **(B)** The white arrow indicates adherent *M. tuberculosis* bacilli at 15 min post infection and the inset shows SEM micrographs of *in vitro* grown *M. tuberculosis* used for infection. **(C)** White arrows indicate *M. tuberculosis* adhering to ARPE-19 cells at 30 min and cell surface changes at the site of attachment of bacilli. The experiment was done in three independent wells and the representative figures are shown.

The adherence of *M. tuberculosis* to RPE cells was initially studied by SEM analysis at 15- and 30- min post-infection. The scanning micrographs (Figure 1) of the uninfected ARPE-19 cells represented an intact monolayer (Figure 1A), while the micrographs of infected ARPE-19 cells showed *M. tuberculosis* adhering to monolayer cells within 15-min of infection (Figure 1B). At 30-min post-infection, cell surface changes and disruption particularly at the site of *M. tuberculosis* attachment were observed (Figure 1C).

**Figure 2:**
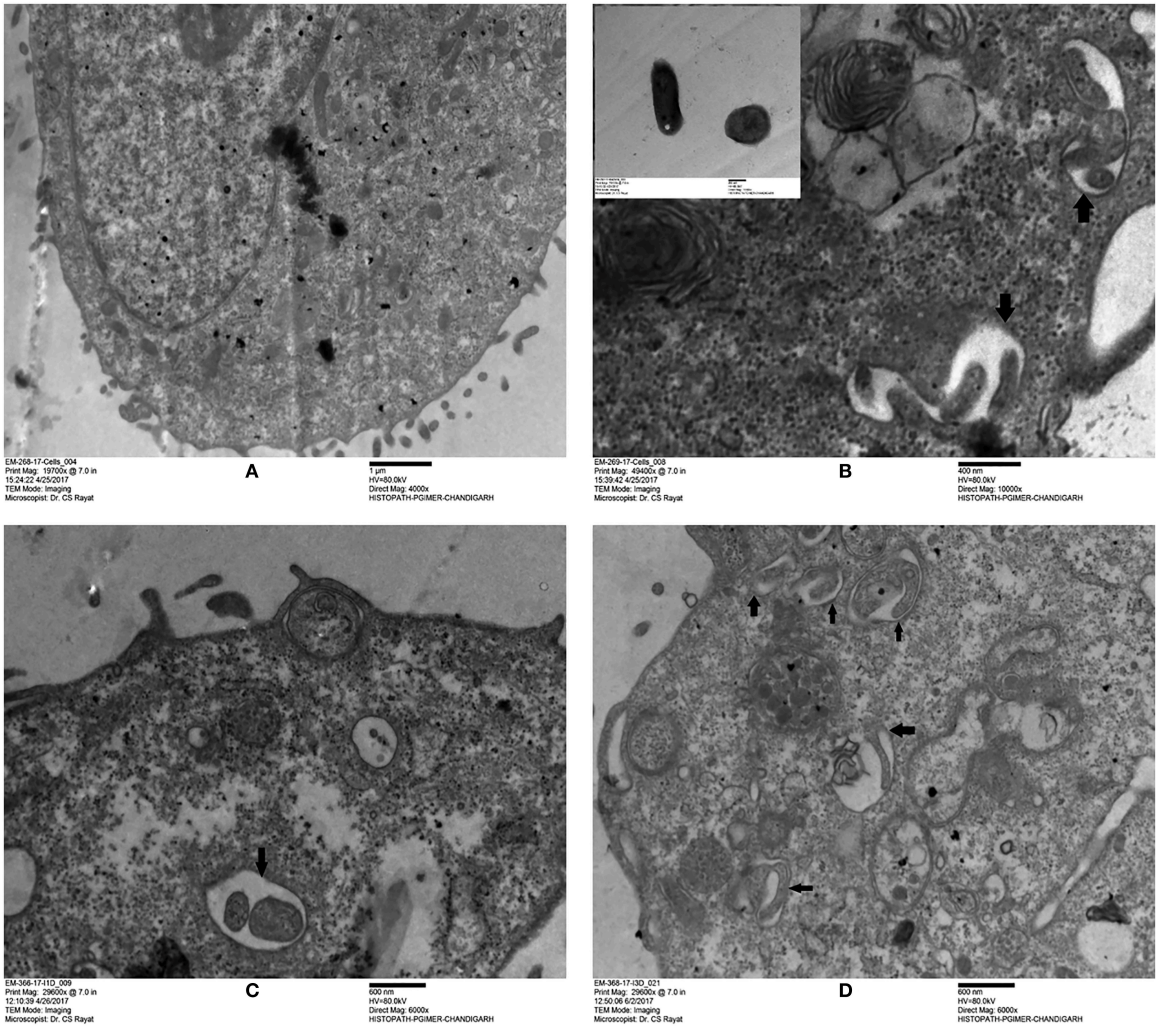
Transmission electron microscopy images of uninfected and *M. tuberculosis* infected ARPE-19 cells. **(A)** Electron micrograph showing a cross-section of uninfected ARPE-19 cells. **(B–D)** Infected ARPE-19 cells after 0- **(B)**, 1- **(C)**, and 3-dpi **(D)**. The figure **(B)** in inset represents *M. tuberculosis* H37Rv cells. Black arrows indicate the vacuolar membrane surrounding the multiple bacilli in ARPE-19 cells. The experiment was done twice and the cells were collected from independent flasks, and the representative figures are shown. dpi, day post infection.

Based on the surface interaction of H37Rv with the ARPE-19 cells, further studies were planned to monitor the invasion of *M. tuberculosis* in ARPE-19 cells using TEM analysis. The mycobacterial cells were visualized as electron dense and electron transparent layer with thick capsular outer layer beyond the cell wall, typical of mycobacterial species as reported earlier (Takade et al., 2003). The uninfected ARPE-19 cell line (0-day) showed a large number of cytoplasmic extensions, high nucleus to cytoplasm ratio, and scanty organelles (Figure 2A). Post amikacin treatment (0-dpi, invasion), multiple bacilli were found in a single vacuolar compartment, indicating internalization/phagocytosis of *M. tuberculosis* by the ARPE-19 cells (Figure 2B). At 1-dpi, bacilli were similarly located inside vacuoles in the cytoplasm (Figure 2C) whereas, at 3-dpi, micrographs showed an increase in the number of vacuolar compartments with multiple bacilli (Figure 2D). Additionally, at 1- and 3-dpi disrupted cellular morphology with degenerated cytoplasm was evident in the infected ARPE cells (Figures 2C,D) while no such changes were observed in the morphology of uninfected ARPE-19 cells at 1- and 3-day (data not shown).

Additionally, the adherence and invasion of *M. tuberculosis* to ARPE-19 cells were quantitatively confirmed by CFU enumeration. The adherence of *M. tuberculosis* to ARPE-19 cells was found to be 22.7% of the initial inoculum [86.2 ± 33.8 (× 10^3^) CFU] within 2 h of incubation with total CFU of 19.7 ± 6.3 (× 10^3^). The percent invasion of *M. tuberculosis* in the RPE cell line as studied after the completion of amikacin treatment (0-dpi) indicated that ARPE-19 cells had taken up 8.4% of the initial inoculum (Table 1).

**Table 1:** *Mycobacterium tuberculosis* adherence, invasion, and replication in *in vitro* model of intraocular tuberculosis.

**RPE cell line**	**Adherence^#^ CFU (× 10^3^) (mean ± SEM) (%)**	**Invasion^$^ CFU (× 10^3^) (mean ± SEM) (%)**	**Multiplication CFU (**×**10**^**3**^**) (mean** ± **SEM)**		
	**2 h**	**0-dpi**	**1-dpi**	**3-dpi**	**5-dpi**
ARPE-19	19.7 ± 6.3 (22.7%)	7.3 ± 2.6 (8.4%)	5.7 ± 0.8	30.2 ± 10.4	47.8 ± 12.1

Values are mean ± standard error of mean (SEM) from 2 independent experiments, having 2 independent wells for each time-point. The ARPE-19 cells were infected with H37Rv at MOI 10:1. The H37Rv inoculum showed 86.2 ± 33.8 (× 10^3^) CFU. The percent (%) adherence (2 h post-infection) and invasion (2 h. post-amikacin treatment, 0 day) was calculated as:

# Percent (%) Adherence = [No. of CFU counts 2 h post-infection] × 100/[Initial inoculum].

$ *Percent (%) Invasion = [No. of CFU counts 2 h post-amikacin treatment (0 day)] × 100/[Initial inoculum]*.

**Figure 3:**
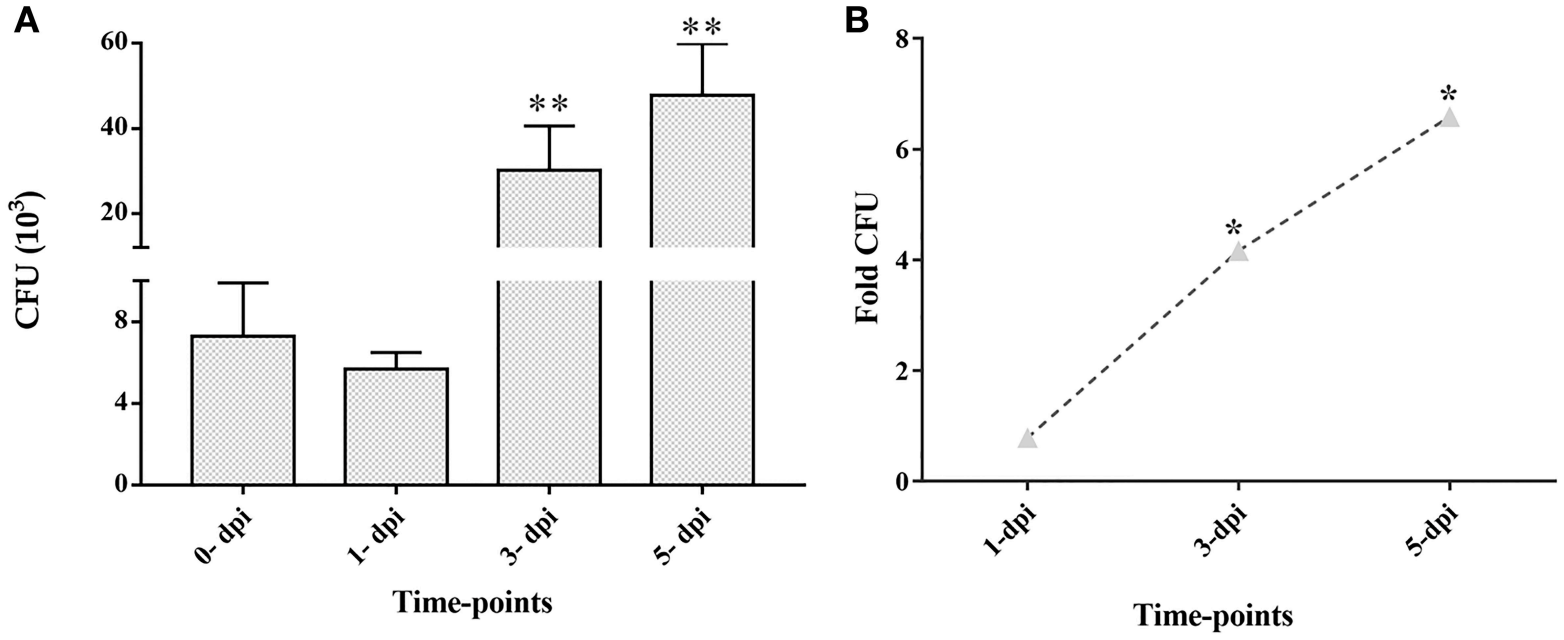
Colony forming units (CFU) and fold CFU of *M. tuberculosis* in ARPE-19 cells at different time-points after infection. ARPE-19 monolayer cells were infected with H37Rv at MOI 10:1. **(A)** CFU counts are mean ± standard error mean (SEM). **(B)** Fold CFU was calculated by dividing the mean CFU at each time-point (1-, 3-, and 5-dpi) by the day 0 CFU. **p < 0.01 and *p < 0.05 in comparison to 0-dpi. Two independent experiments were performed and cells were infected in duplicate for each time-point. dpi, day post-infection.

CFU enumeration at later time points of infection demonstrated the intracellular multiplication of *M. tuberculosis* in ARPE-19 cells. The infected ARPE-19 cells showed 5.7 ± 0.8 (× 10^3^) CFU at 1-dpi, 30.2 ± 10.4 (× 10^3^) CFU at 3-dpi, and 47.8 ± 12.1 (× 10^3^) CFU at 5-dpi (Table 1 and Figure 3A). The increase in intracellular *M. tuberculosis* growth over time was calculated by an increase in fold CFU at 1-, 3-, and 5-dpi in comparison to day 0.The bacterial replication was found to be increased by four-fold at 3-dpi while it was increased up to 6.5-fold at 5-dpi, and this increase in bacterial growth was significant (*p* < 0.05) in comparison to 0-dpi (Figure 3B).These results indicate that *M. tuberculosis* H37Rv adheres, invades, and utilizes RPE cells for intracellular replication.

#### Cytotoxicity of *M. tuberculosis* to retinal pigment epithelial cell line

**Figure 4:**
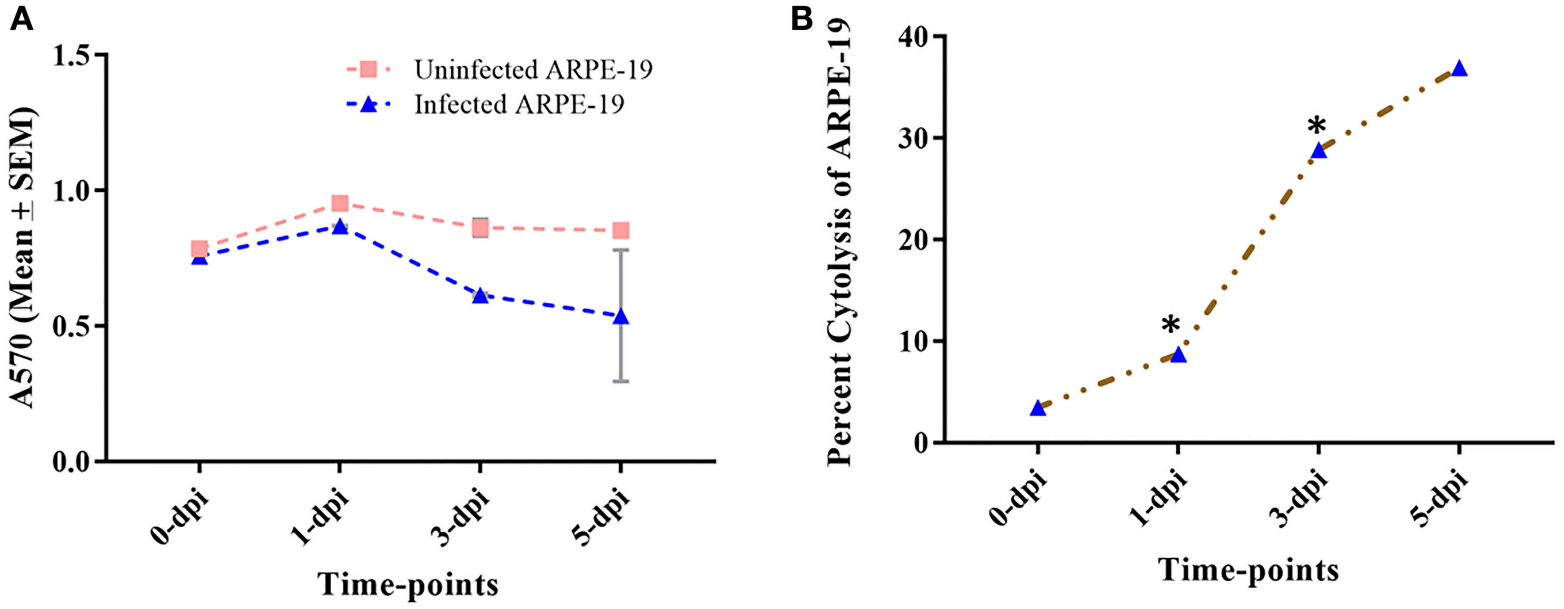
Percent cytotoxicity caused by *M. tuberculosis* to ARPE-19 cells. **(A)** Mean ± SEM O.D. values at 570 nm (A570) of uninfected and infected ARPE-19 cells at different time-points (0-,−1-, 3-, 5-dpi). **(B)** Cytolysis of cells was quantified using MTT dye at different time-points. *p < 0.05 in comparison to 0- dpi. The percent (%) cytotoxicity was calculated as = (Mean O.D. of control cells - Mean O.D. of infected cells)**/**Mean O.D. of control cells × 100. dpi, day post infection; SEM, standard error of the mean.

To further confirm the observations obtained from electron microscopy and to determine the effect of intracellularly multiplying bacilli on the viability of ARPE-19 cells, an MTT assay was performed. The mean optical density (O.D.) obtained at 570 nm (A570) for uninfected and infected ARPE-19 cells at different time-points are plotted in Figure 4A. A significant decrease in A570 at the start of intracellular replication (1 dpi; *p* < 0.05) with further decrease at 3 dpi, as compared to the uninfected cells (*p* < 0.05), indicated a loss of cell viability in the infected cell line. Under similar conditions, A570 was maintained in case of uninfected cells till 5 dpi indicating no significant change in cell viability. In terms of percent cytolysis, *M. tuberculosis* caused 3.5, 8.8, 28.8, and 36.9% loss in ARPE-19 cell viability at 0−, 1−, 3−, and 5-dpi, respectively, with significant loss of cells occurring at 1-dpi (*p* < 0.05) and 3-dpi (*p* < 0.05) in comparison to their respective controls (Figure 4B).

### Intracellular fate of *M. tuberculosis* in retinal pigment epithelial cells

As the ability of mycobacteria to survive in host cells is attributed to colocalization with endosomal/lysosomal compartments (Hasan et al., 1997; Huynh et al., 2007), after confirming the adherence, invasion, and intracellular multiplication of *M. tuberculosis* in RPE cells, we were further interested to determine the intracellular fate of *M. tuberculosis*. For this purpose, the ARPE-19 cells were infected with PKH26-labeled *M. tuberculosis* bacilli followed by confocal microscopic analysis at 0-, 1-, and 3-dpi.

#### *Mycobacterium tuberculosis* colocalizes with LAMP-1and LAMP-2 in ARPE-19 cell line

**Figure 5:**
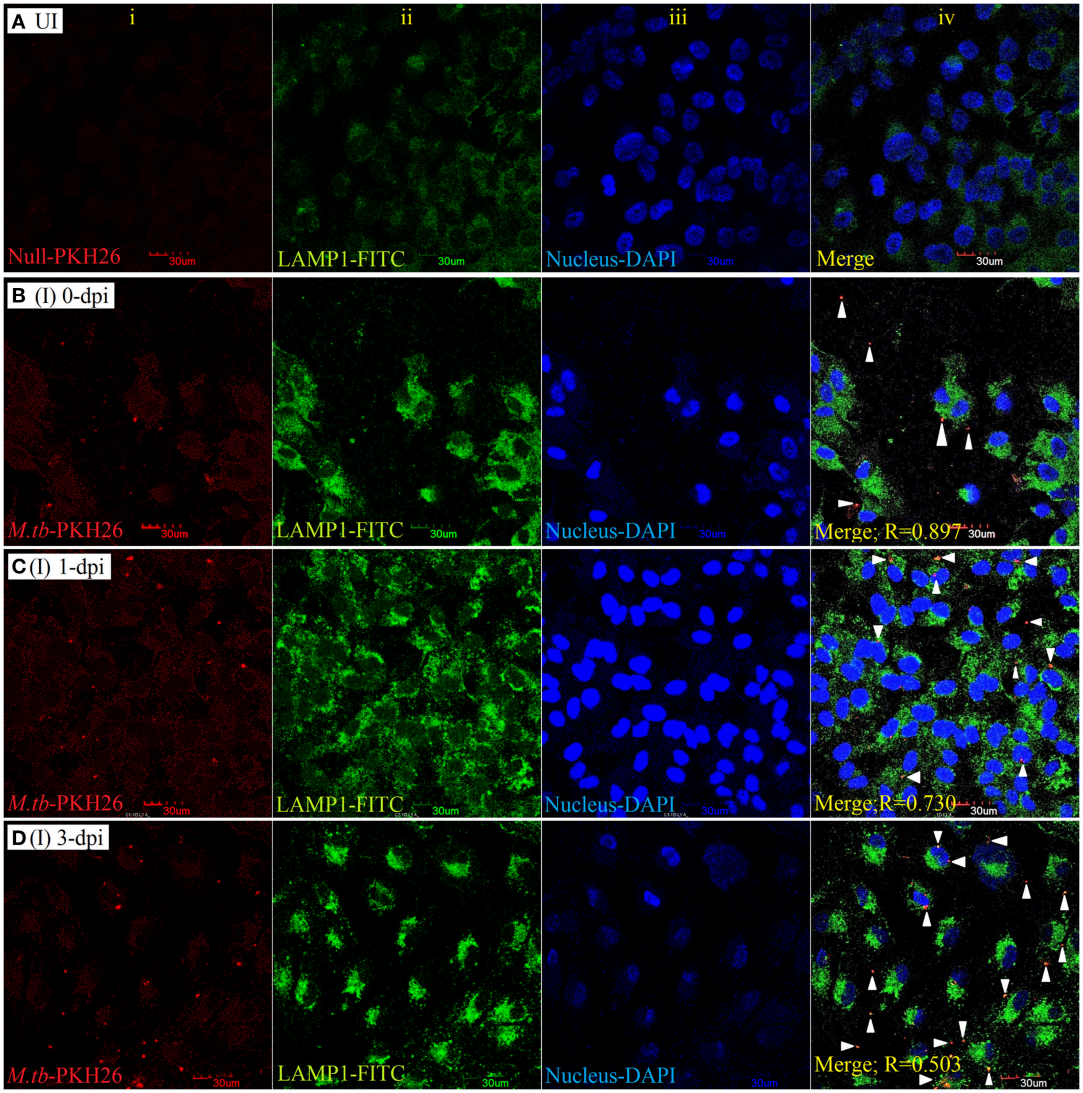
LAMP-1 staining for lysosomal localization of *M. tuberculosis* in RPE (ARPE-19) cells. Confocal micrographs of uninfected (UI) **(A)** and infected (I) ARPE-19 cells (MOI 10:1) at 0-dpi **(B)**, 1-dpi **(C)**, and 3-dpi **(D)** showing staining for (i) *M. tuberculosis* labeled with PKH26 dye (red channel); (ii) LAMP-1 antibody using secondary FITC-IgG (green channel); (iii) DAPI for nuclei (blue channel). In infected ARPE-19 cells (rows **B–D**; column iv), white arrowheads show merging of two channels (red and green) and the Pearson's R coefficient for the colocalization of *M. tuberculosis* with LAMP-1. Zero is no colocalization, and 1 means perfect colocalization. Dpi, day post-infection; UI, uninfected; I, infected.

**Figure 6:**
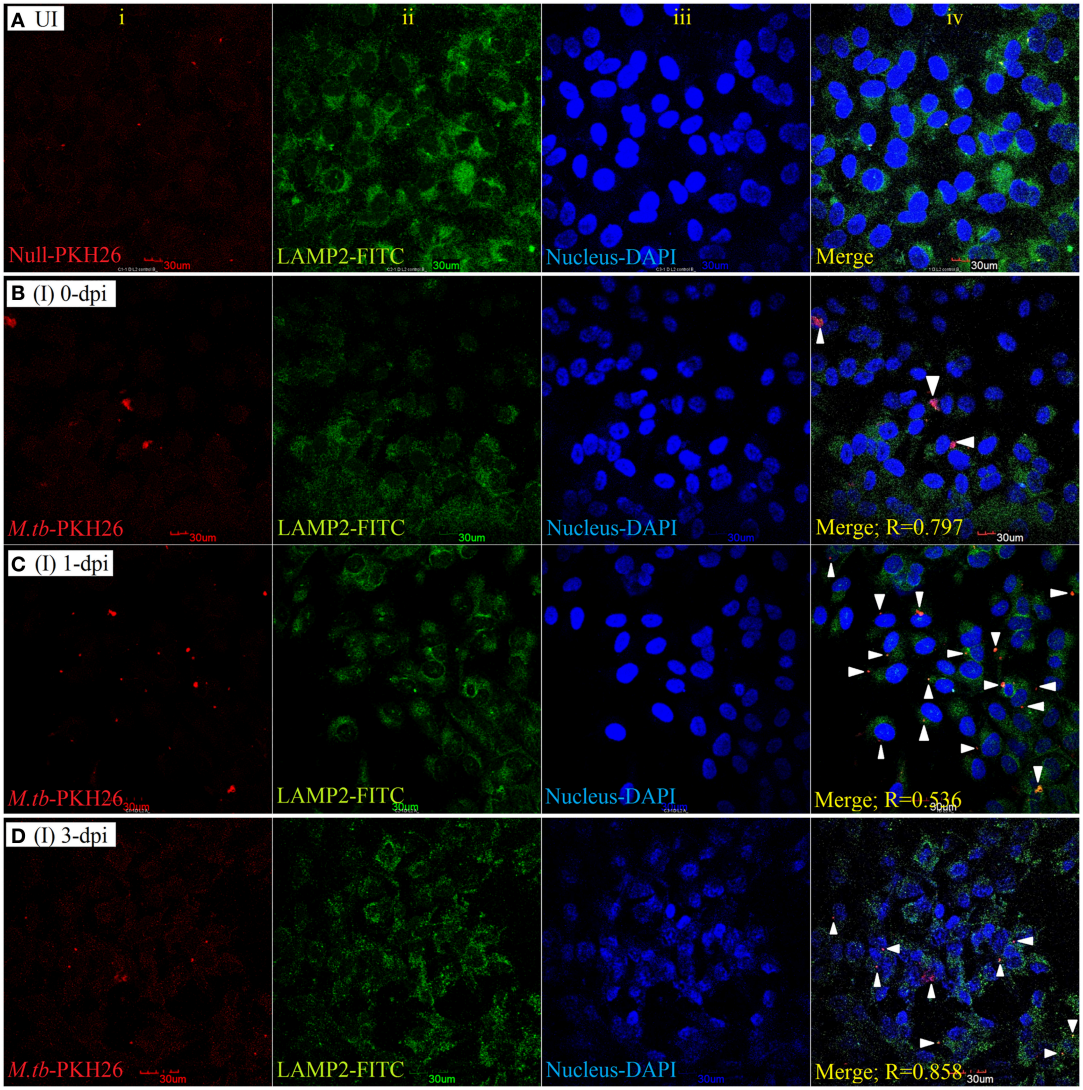
LAMP-2 staining for lysosomal localization of *M. tuberculosis* in RPE (ARPE-19) cells. Confocal micrographs of uninfected (UI) **(A)** and infected (I) ARPE-19 cells (MOI 10:1) at 0-dpi **(B)**, 1-dpi **(C)**, and 3-dpi **(D)** showing staining for (i) *M. tuberculosis* labeled with PKH26 dye (red channel); (ii) LAMP-2 antibody using secondary FITC-IgG (green channel); (iii) DAPI for nuclei (blue channel). In infected ARPE-19 cells (rows **B–D**; column iv), white arrowheads show merging of two channels (red and green) and the Pearson's R coefficient for the colocalization of *M. tuberculosis* with LAMP-2. Zero is no colocalization, and 1 means perfect colocalization. Dpi, day post-infection; UI, uninfected; I, infected.

To examine the localization of *M. tuberculosis* to late endosomes/lysosomes, we used antibodies against LAMP-1/LAMP-2 proteins, the most abundant proteins in lysosomal membranes. Post amikacin treatment (0-dpi), the PKH26-labeled bacilli showed partial colocalization with both LAMP-1 (Figure 5) and LAMP-2 (Figure 6) marker proteins (green: LAMP-1/LAMP-2 and red: *M. tuberculosis*) as revealed by an analysis performed using Coloc2-ImageJ plugin that provided Pearson's coefficient (R) values of 0.897 and 0.797, respectively (where zero is no colocalization, 1 means perfect colocalization). With LAMP-1, the R values corresponded to 0.730 and 0.503 at 1- and 3-dpi, respectively (Figure 5), whereas with LAMP-2, the *R*-values corresponded to 0.536 and 0.858 at 1- and 3-dpi, respectively (Figure 6). A number of intracellular bacilli were also found in free form, without any colocalization with LAMP-1/LAMP-2 at 3-dpi (Figures 5, 6; row D, column iv), suggesting the survival and high fold replication of *M. tuberculosis* in the cytoplasm of ARPE-19 cells. Overall, these results suggest that following invasion (0-dpi), *M. tuberculosis* gets recruited to the endocytic pathway and partially colocalizes with the late endosome/lysosomes markers during its replication at 1- and 3-dpi within the RPE cells. A similar type of intracellular fate of *M. tuberculosis* has also been reported in other professional and non-professional phagocytic cells (Hasan et al., 1997; Huynh et al., 2007).

### Transcriptional profile of intracellular *M. tuberculosis* in *in vitro* model of intraocular tuberculosis

The decrease in viable CFU count at 1-dpi in comparison to 0-dpi (Figure 3A) followed by a steady increase in intracellular CFU load till 5-dpi (Figure 3B) indicates the adaptation of few intracellular bacilli after initial infection of ARPE-19 cells. This survival and adaptation of *M. tuberculosis* in an *in vitro* RPE model of infection could be due to altered bacterial physiological behavior that could be inferred through global transcriptome analysis (Rohde et al., 2012). Additionally, such analysis could also be significant for identifying the metabolic checkpoints which are involved in successively averting the host hostile environment and may be exploited for therapeutics for avoiding the replication of *M. tuberculosis* (Du et al., 2016).

#### Transcriptional signatures of *M. tuberculosis* inside RPE cells

**Figure 7:**
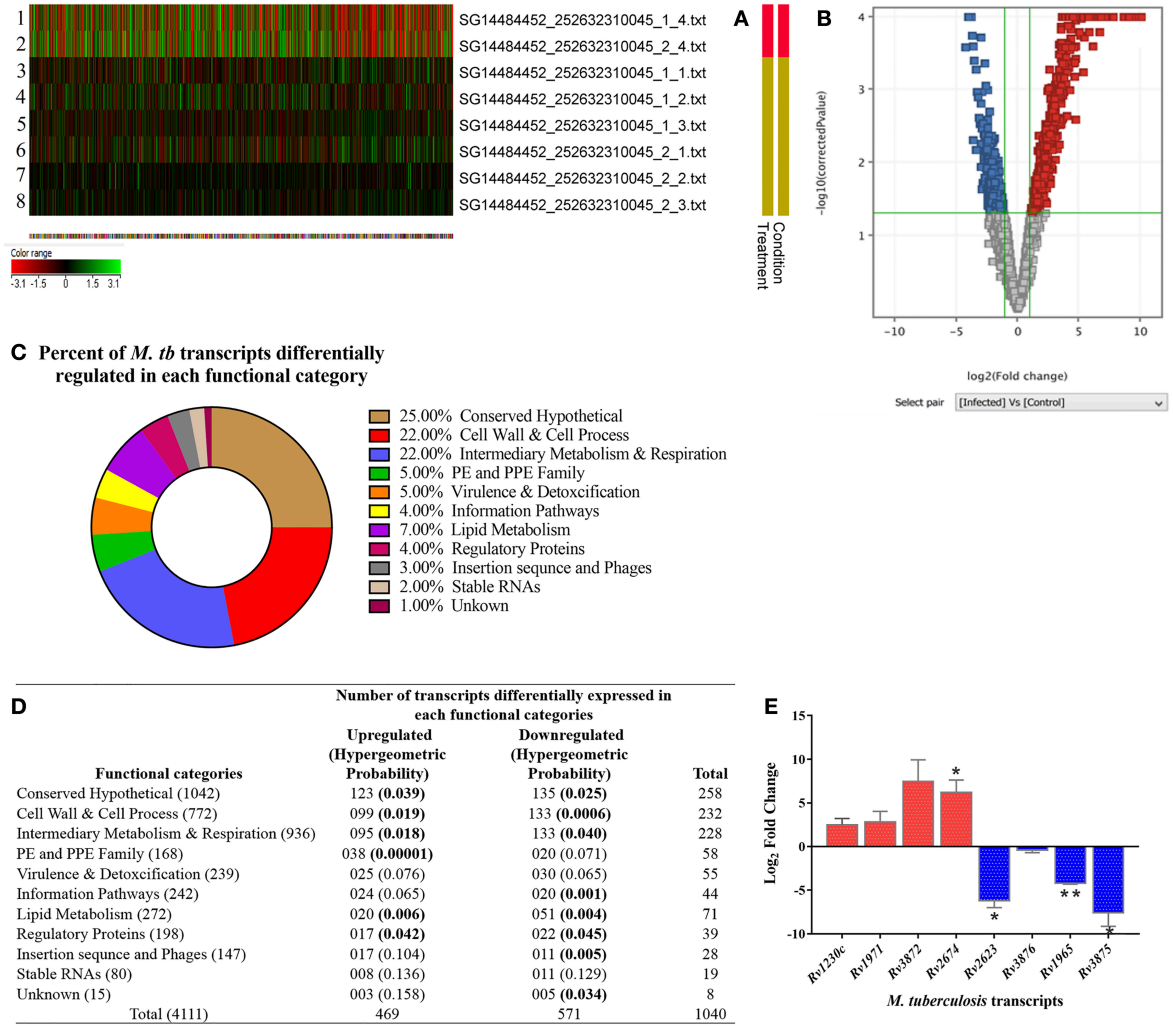
Transcriptome analysis of intracellular *M. tuberculosis* in *in vitro* model of intraocular tuberculosis. **(A)** The heat map shows the 2 control samples (*in vitro M. tuberculosis* as inoculum used during infection; rows 1, 2) and 6 test samples (three biological replicates, each with two technical replicates. Rows 3-5 is biological and row 6-8 is technical set) from 3 independent experiments. **(B)** Volcano plot shows the upregulated genes (blue boxes), the downregulated genes (red boxes), and the unchanged genes (gray boxes). The x-axis denotes log2 fold change whereas the Y-axis denotes the log_10_ of *p*-values. **(C)** The pie chart shows the percentage of differentially regulated genes in each functional category as per the TubercuList. **(D)** The table shows the number of genes upregulated and downregulated in each functional category. The transcripts at 2.0-fold change cut-off were considered to be differentially regulated at *p* < 0.05. To avoid the false positive selection of transcripts, multiple testing correction was performed using Benjamini–Hochberg procedure in Gene Spring software. To check the statistical number of genes differentially regulated in each functional category, Hypergeometric probability (value in parentheses) was applied and bold values indicate the statistically significant at *p* < 0.05. **(E)** Validation of microarray data on qRT-PCR by analyzing the relative expression of 9 genes (5 upregulated and 4 downregulated) in two independent experiments (3-dpi), with technical replicates as compared to *in vitro* grown *M. tb* H37Rv. 16s rRNA was used as reference gene for normalization. Y-axis values (Log_2_ fold change) of ≥1 indicate upregulation and values ≤ -1 indicate down-regulation. Each bar represents mean ± SD values for each of the genes with two biological and two technical replicates. *p < 0.05; **p < 0.01 by student's *t-*test.

We analyzed *M. tuberculosis* transcriptional signatures by whole genome microarray of intracellular bacilli at 3-dpi in the ARPE-19 cells. The heat map shows the signal intensities in H37Rv control (rows 1, 2) and infected (rows 3–8) experimental sets (Figure 7A). Hierarchical clustering of microarray data demonstrated that the infected and control samples were overlapping to their respective technical sets, and the principal components analysis further confirmed the hierarchical clustering as the correlation coefficient value of each data set were similar and were thus treated as experimental replicates (Supplementary Figure 1). Among all the genes encoded by *M. tuberculosis* genome, the transcripts corresponding to 1,040 genes were found to be differentially regulated (cut off 2.0) in comparison to H37Rv control. Out of these differentially regulated transcripts, 469 transcripts were found to be upregulated whereas 571 transcripts were downregulated. The volcano graph (Figure 7B) shows the upregulated (blue box), downregulated (red box), and unchanged (gray boxes) transcripts. The upregulation and downregulation of differentially regulated transcripts were in the range of +1.15 to +10.1 Log_2_ fold change and −4.2 to −1.15 Log_2_ fold change, respectively (Supplementary Table 2; GEO accession number # GSE115292). Comparison of differentially regulated transcripts with the earlier reported data (Jain et al., 2006; Rohde et al., 2012; Ryndak et al., 2015) from other *in vitro* models of *M. tuberculosis* infection indicated that many of these transcripts were uniquely regulated in *in vitro* model of IOTB (Supplementary Table 2).

The differentially expressed *M. tuberculosis* transcripts in RPE cells were functionally categorized and organized as per the TubercuList database (Lew et al., 2011). The highest percentage of transcripts that were differentially regulated encode for proteins corresponding to the categories of conserved hypothetical (25%), cell wall and cell process (22%), and intermediary metabolism and respiration (22%). Around 4–7% of the differentially regulated transcripts belonged to the PE and PPE families, regulatory proteins, virulence and detoxification, information pathways, or lipid metabolism. Remaining 1–3% transcripts belong to insertion sequences, phages, or stable RNAs functional categories (Figure 7C). Each functional group contained a different number of upregulated and downregulated transcripts (Figure 7D). The hypergeometric test (*p* < 0.05) was applied to determine the functional categories that were enriched in differentially expressed transcripts (Figure 7D) (Leong and Kipling, 2009; Qureshi and Sacan, 2013; Sharma et al., 2017). The annotated list of identified *M. tuberculosis* transcripts with a potential role in invasion, transcriptional mechanism, environmental adaptation, and survival inside RPE cells at 3-dpi has been provided in Table 2 according to their functional categories as sorted by the TubercuList database (http://tuberculist.epfl.ch).

**Table 2:** Predicted/known functions of *M. tuberculosis* gene products whose transcripts were upregulated at time of replication of bacteria inside ARPE-19 cells, an *in vitro* model of intraocular tuberculosis.

**Functional categories**	**Known/Predicted functions^#^**	**Genes**
Virulence and Detoxification	Host cell invasion	*Rv1971, Rv0169, Rv0592*, and *Rv1967*
	Adaptive process	*Rv1026*
	Stress condition	*Rv2028c*
	Survival	*Rv0169, Rv1026, and Rv2028c*
Regulatory Proteins	Transcriptional mechanism	*Rv0823c, Rv3160c, Rv2621c, Rv1963c, Rv1990c, Rv3416, Rv1556, Rv3164c, Rv0232, Rv1129c, Rv2324, Rv3183*, and *Rv3833*
	Environmental adaptation and intracellular growth	*Rv0902c*
PE and PPE families of proteins	Promotes the bacterial attachment to host cells	*Rv1759c*
Intermediary Metabolism and Respiration	Intracellular survival	*Rv2672 and Rv1755c*
	Cellular metabolism	*Rv0044c, Rv3712, Rv0197, Rv0561c, Rv3329, Rv2962c, Rv1723*, and *Rv0245*
	Repair enzyme	*Rv2674*
Information Pathways	Translation activity	*Rv2056c, Rv3459c, Rv3456c, Rv3462c, Rv0979A*, and *Rv1630*
	Replication of bacteria	*Rv3721c*
Cell Wall and Cell Process	Regulating the bacterial replication under stress conditions or modulating the host immune responses	*Rv1884c* and *Rv2450c*
	Survival	*Rv2235*
Conserved Hypothetical	Survival/Virulence	*Rv2844, Rv0775, Rv2541, Rv3566A Rv1518, Rv1540, Rv0323c, Rv2414c Rv1752, Rv3654c, Rv1993c, Rv1907c Rv2828A, Rv2803, and Rv0381c*

# *Genes product functions as reported in TubercuList (http://tuberculist.epfl.ch)*.

#### Correlating physiological state and the transcriptional signatures of *M. tuberculosis* in *in vitro* model of IOTB

To further study the successful adaptation, survival, and replication of *M. tuberculosis* in the host RPE cells, we analyzed the involvement of altered transcripts in *M. tuberculosis* metabolic pathways. Identifying the genetic network of metabolism is important in understanding the physiology and pathogenesis of *M. tuberculosis* in a hostile host environment (Rohde et al., 2012; Warner, 2014; Du et al., 2016). Using BioCyc database (Karp et al., 2017) we identified the changes in metabolic pathways and defined the transcriptional signatures involved in the modulation of cellular functions such as biosynthesis, energy, central dogma, regulation, cellular process, cell exterior, and response to a stimulus. The complete SmartTable (BioCyc dashboard display) of cellular functions along with the defined transcripts with Log_2_ fold change can be visualized using the following link (https://biocyc.org/dashboard/dashboard.html?st=biocyc13-29738-3741434995). Overall, it was observed that the intracellular *M. tuberculosis* in RPE cells upregulates the transcripts from the cellular process and cell exterior classes as their protein products are involved in host interaction, adhesion, cell cycle, and biogenesis/organization of cell wall, thus suggesting the adaptation and symbiosis of mycobacteria with the host RPE cells. The upregulation of transcripts encoding for the proteins involved in energy metabolism suggests the survival of bacteria inside the RPE cells. The upregulation of transcripts whose protein products are involved in biosynthesis of amino acid, fatty acid and lipids, carbohydrates, DNA, RNA, and proteins demonstrated the growth and replication (Supplementary Table 3). Thus, the transcriptional profile of *M. tuberculosis* during the replication stage after the adaption in host RPE cell demonstrates the alteration in transcripts controlling the physiological behavior of *M. tuberculosis* inside the RPE cells for its survival and intracellular replication, thus leading to productive infection.

#### Validation of *M. tuberculosis* transcripts identified in *in vitro* model of intraocular tuberculosis

To validate the microarray data, we analyzed the expression of the top-most upregulated (*Rv1230c*) and downregulated (*Rv2623*) transcripts along with few other transcripts from the upregulated (*Rv1971, Rv3872*, and *Rv2674*) and downregulated (*Rv3876, Rv1965*, and *Rv3875*) categories by qRT-PCR. Quantitative RT-PCR showed similar upregulation and downregulation profile of the transcripts as detected on a microarray platform. *Rv1230c, Rv1971, Rv3872*, and *Rv2674* were 2.5, 2.8, 7.4, and 6.5 Log_2_ fold upregulated, respectively, while *Rv2623, Rv3876, Rv1965*, and *Rv3875* were −6.2, −0.4, −4.2, and −7.6 Log_2_ fold downregulated, respectively (Figure 7E). Altogether, the qRT-PCR results were in concordance with the microarray data.

### *Mycobacterium tuberculosis* transcripts in vitreous samples from intraocular tuberculosis patients

Three *M. tuberculosis* transcripts showing upregulated expression in infected ARPE-19 cells were also tested qualitatively by RT-PCR for *in vivo* expression in human vitreous samples. Based on the C_T_ value (<35) and specific melt curves as explained in methodology, *Rv1230c* was identified in 6/7 confirmed human IOTB samples and in all the three possible IOTB cases; whereas, *Rv1971* was detected in 6/7 confirmed and in 1/3 possible IOTB cases (Table 3). However, *Rv3872* was not detected in any of the human IOTB samples. For all the three genes, H37Rv RNA showed C_T_ value <35 and specific product melt curves, whereas in case of all the negative control no C_T_ and transcript-specific melt curve was obtained.

**Table 3:** *Mycobacterium tuberculosis* transcripts in vitreous samples from intraocular tuberculosis patients.

**Samples categories**	**Sample ID**	***Rv1230c***	***Rv1971***
Possible IOTB	H-IOTB-S4	D+	–
	H-IOTB-S5	R+	–
	H-IOTB-S9	D+	D+
Confirmed IOTB	H-IOTB-S11	–	–
	H-IOTB-S12	D+	D+
	H-IOTB-S13	D+	D+
	H-IOTB-S14	R+	D+
	H-IOTB-S15	D+	D+
	H-IOTB-S16	R+	R+
	H-IOTB-S17	R+	D+

*Human vitreous samples from two different categories of IOTB were examined for M. tuberculosis transcripts corresponding to Rv1230c, Rv1971, and Rv3872 genes using H37Rv (as a positive control). Transcripts which showed C_T_ value in RT+ sample were denoted as R+ (as the C_T_ was due to cDNA synthesized from RNA), while early C_T_ value in RT- of the same sample was denoted as D+ (as the C_T_ was due to the presence of DNA). Rv3872 is negative for all clinical samples at both RNA and DNA levels. IOTB, intraocular tuberculosis; cDNA, complementary deoxyribonucleic acid; RT, reverse transcriptase; RT-PCR, real-time polymerase chain reaction; H-IOTB, all clinical samples identity number. +, Positive; –, Negative*.

## Discussion

An *in vitro* model of IOTB was established using the RPE cell line ARPE-19. Retinal pigment epithelial cells are the primary barrier cells in the eye to counter any invading pathogen and have been chosen as an experimental model in other diseases also, such as endophthalmitis caused by *Bacillus cereus*, to understand the host-pathogen interactions (Callegan et al., 2007). Supporting the earlier reported clinical and histopathological evidence for localization of AFB in RPE cells (Rao et al., 2006), the experimental studies with the ARPE-19 cell line in the present study also demonstrated that *M. tuberculosis* can utilize these cells for its survival. The scanning electron micrographs of the ARPE-19 cell line infected with *M. tuberculosis* showed the adherence of *bacilli* to these cells along with morphological cell surface changes (Figure 1). Similar morphological changes have been reported in the airway and alveolar epithelial cells after the adherence and uptake of *M. tuberculosis* (García-Pérez et al., 2003, 2008, 2012; Hall-Stoodley et al., 2006). These surface changes in non-professional phagocytic cells usually triggered by bacterial proteins (Hall-Stoodley et al., 2006; Ham et al., 2011; Vir et al., 2014) are considered as the mechanism evolved for the uptake and invasion of the pathogens such as *M. tuberculosis, Salmonella typhimurium*, and *Listeria monocytogenes* (Garcia-del Portillo and Finlay, 1994; Menon et al., 2003). Following the invasion, the intracellular localization of *M. tuberculosis* in the ARPE-19 cell line was monitored by TEM analysis (Figure 2) which showed *M. tuberculosis* bacilli surrounded by vacuolar membranes in the cytoplasm of ARPE-19 cells (Figure 2B). The presence of bacteria within the vacuoles in the RPE cell lines was in concordance with a similar study conducted in A549 alveolar epithelial cell line where multiple bacilli within the vacuoles were seen (Mehta et al., 1996). At 3-dpi, the RPE cells showed numerous vacuoles containing multiple bacilli, degenerated cytoplasm with disrupted morphology (Figure 2D) in comparison to 0-dpi (Figure 2B), suggesting that the increased mycobacterial load is cytotoxic to RPE cells (Figure 4B). The adherence and invasion along with multiplication of *M. tuberculosis* in ARPE-19 cells were also quantified using CFU enumeration which showed a significant percentage of *M. tuberculosis* adhering to and invading the RPE cells (Figure 3A and Table 1). Further, an increase in fold CFU of intracellular bacilli observed till 5-dpi (Figure 3B) confirmed the intracellular replication similar to that reported earlier in alveolar non-professional (Mehta et al., 1996; Dobos et al., 2000) and professional phagocytic cells (Sharma et al., 2012; Scharn et al., 2016). Thus, it is evident that the RPE cells, the non-professional phagocytic cells in the eye, provide a permissive habitat to virulent *M. tuberculosis* like other professional phagocytic cells (Russell et al., 2009). With the significant increase in intracellular growth, a loss in cellular viability of infected host cells was seen at 3-dpi and at 5-dpi (Figure 4B) in comparison to uninfected cells. Earlier, in A549 cells also, it has been reported that cytotoxicity at 3- and 5-dpi was related to the cytotoxic phenotype of increased mycobacterial growth (Mehta et al., 1996; Dobos et al., 2000). The loss in cellular viability of infected cells during *M. tuberculosis* replication in professional phagocytic cells is considered as a mechanism so that the dead cells are utilized as a bait for new neighboring cells for the dissemination of infection (Cambier et al., 2014; Mahamed et al., 2017). However, no such mechanism is known yet in the case of RPE cells.

The canonical pathway (endosomal-lysosomal trafficking) of intracellular fate of *M. tuberculosis* has already been elucidated via various *in vitro* models of professional (macrophages, neutrophils, monocytes, and dendritic cells) (López de Armentia et al., 2016) and non-professional phagocytic cells (epithelial, endothelial cells) (Baltierra-Uribe et al., 2014; López de Armentia et al., 2016). *Mycobacterium tuberculosis* in both types of phagocytic cells is known to block the final step of the maturation process involving fusion of phagosomes with lysosomes, thus inhibiting the formation of phagolysosome and resulting in its intracellular multiplication and survival (Seto et al., 2012). It has also been established that *M. tuberculosis* inhibits the fusion of lysosomes with phagosomes through the selective exclusion of the GTPase Rab7 and lysosomal-associated membrane protein 1 (LAMP-1) or LAMP-2 coupled with the retention of Rab5 on the phagosome (Via et al., 1997; López de Armentia et al., 2016). If these mechanisms are employed by tubercle bacilli for the intracellular survival in RPE cells also is still not known. The co-localization of PKH26-labeled *M. tuberculosis* with LAMP-1 (Figure 5) and LAMP-2 (Figure 6) during intracellular replication at 1- and 3-dpi was to lesser extent as compared to 0-dpi in ARPE-19 cell line. Decrease in colocalization with late endosomal markers during the multiplication stage of *M. tuberculosis* in the RPE cells suggests the deceleration of phagosomal maturation. Similarly, in human monocyte-derived dendritic cells and macrophages, tubercle bacilli were found translocating from phagolysosomes into the cytosol 2 days after infection (Van der Wel et al., 2007). This could be due to an attempt at multiplying *M. tuberculosis* for its cytosolic localization (Van der Wel et al., 2007), as bacteria in the cytosol are considered to have a superior capacity for overpowering the host cellular autophagy, a lysosomal degradation pathway for cytoplasmic materials (Jamwal et al., 2016). Intracellular fate of *M. tuberculosis* in non-professional phagocytic cells is dependent on more than one endocytic routes (Baltierra-Uribe et al., 2014). A detailed labeling protocol for early and late endosomes with various Rab family proteins will further help to elucidate the trafficking events of *M. tuberculosis* in RPE cells more appropriately.

Delineating the pathogen and/or host genetic factors is not only important for understanding the co-evolution of host-pathogen but also to have an insight in the capabilities of the pathogens in successively modulating the hostile host environment into a favorable niche (Reiling et al., 2018). Studying the transcriptional signatures of pathogens in an appropriate host is therefore known to aid in studying the disease pathogenesis (Ward et al., 2010). Therefore, we analyzed the mycobacterial transcriptional signatures in *in vitro* ARPE cell line model of IOTB in an attempt to understand the *M. tuberculosis* adaptation and survival in host RPE cells. The microarray technology was utilized as it is a well-established tool to study the whole-genome transcriptome leading to the availability of extensive information in the field of functional genomics (Nookaew et al., 2012). The identified transcripts were functionally categorized using the TubercuList database (Supplementary Table 2). The role of protein products of these transcripts in the physiological status of intracellularly replicating *M. tuberculosis* inside host environment was further described by studying the modulation in various metabolic pathways (through BioCyc database; Supplementary Table 3). Out of 1,040 differentially expressed transcripts by intracellularly replicating *M. tuberculosis* in ARPE-19 cells, we have primarily focused on those transcripts whose products (proteins) have known or hypothetical functions in the invasion, adaptation, survival, and replication in the host environment. The functional annotation of these transcripts is based on the studies carried out in other experimental conditions (*in vivo* or *in vitro*) of *M. tuberculosis* infection and such commonly expressed transcripts have been considered as hallmark transcriptional response of *M. tuberculosis* (Talaat et al., 2004; Rohde et al., 2012), thus supporting the validity of transcripts identified in the present study. In addition, many transcripts were uniquely altered in the *in vitro* model of IOTB (Supplementary Table 2) as these transcripts have not been reported earlier in other *in vitro M. tuberculosis* infection models (Jain et al., 2006; Rohde et al., 2012; Ryndak et al., 2015). It is important to understand the role of these uniquely identified transcripts in the pathogenesis of IOTB and could be exploited for new diagnostics/therapeutics for IOTB.

Bacterial pathogens are known to subvert the hostile host environment by regulating its transcription regulatory network which primarily includes the activation of two-component system and/or alternative sigma factors leading to modulation in the bacterial metabolism and physiology (Leistikow et al., 2010; Flentie et al., 2016). We found that *Rv2028c* transcript (from Rv2028-Rv2031 operon) was upregulated and its protein is known to regulate the different metabolic pathways and sub-cellular processes like the two-component system (DevS-DevR) under stress condition (Mushtaq et al., 2015). Another upregulated transcript of Rv*0902c* is also a part of two-component regulatory system (Rv0903c-Rv0902c) and is known to play a role in environmental adaptation and intracellular multiplication inside murine (Chandolia et al., 2014) and human (Graham and Clark-Curtiss, 1999; Haydel and Clark-Curtiss, 2004) macrophages and also controls mycobacterial metabolism and viability in professional phagocytic cells (Haydel et al., 2012). Additionally, the upregulation of 2 sigma factors transcripts of *sigJ (Rv3328c)* and *sigM (Rv3911)* and another possible sigma factor *Rv1364c* transcripts were observed during *M. tuberculosis* intracellular replication in ARPE-19 cells. Upregulation of ribosomal (*Rv2056c, Rv3459c, Rv3456c, Rv1630, Rv0720, Rv0979A, Rv1642*, and *Rv0700*), transcriptional (*Rv0823c, Rv1994c, Rv3765c, Rv3160c, Rv1963c, Rv3416, Rv0232, Rv0602c, Rv1129c, Rv2324, Rv3183*, and *Rv3833*), and translation initiation (*Rv3462c*) transcripts whose protein products have a role in RNA metabolism of mycobacteria (Supplementary Table 3) also suggested the active replicative state of *M. tuberculosis* inside the RPE cells. All these transcripts of sigma factors, ribosomal and translation regulators belonging to information and regulatory protein categories (Supplementary Table 2) were found to be uniquely regulated in the *in vitro* model of IOTB. However, among the transcriptional regulators, *Rv0823c, Rv3416*, and *Rv1129c* transcripts have earlier been defined in the primary macrophage model also (Rohde et al., 2012) and the *Rv3833* transcript is known to be upregulated in another immune privileged *in vitro* central nervous system-TB model (Jain et al., 2006). Rv1129c has been identified as a transcription factor that is directly involved in the regulation of enzymes of methylcitrate cycle for cholesterol consumption during intracellular growth (Griffin et al., 2012) and Rv3833 is a member of AraC transcriptional regulator family which regulates the genes involved in carbon metabolism, stress responses, and virulence (Martin and Rosner, 2001; Frota et al., 2004).

Further, transcripts encoding proteins with a role in the adaptive process (*Rv1026*) or in stress (*Rv3667*) condition (Lew et al., 2011) were also upregulated in *M. tuberculosis* infected RPE cells. *Rv1026* has been defined with a key role in maintaining the *M. tuberculosis* intracellular growth and persister-like features inside macrophages, thus leading to antibiotic tolerance (Chuang et al., 2015). Rv3667 is known to aid in reducing the metabolic stress experienced by bacterium inside the macrophages (Lee et al., 2013). To further maintain the balance between several stress and physiological conditions, a multifaceted integrated network known as the MprAB system is involved (He et al., 2006; Pang et al., 2007). This system regulates the diverse subnetwork of gene panel encoding for NADH dehydrogenase complex (Du et al., 2016). Five transcripts of probable NADH dehydrogenase complex (*Rv3153*: *nuoI, Rv3147: nuoC, Rv3149: nuoE, Rv3154: nuoJ*, and *Rv3155*: *nuoK*) involved in aerobic respiration were upregulated (Supplementary Table 3) during intracellular *M. tuberculosis* replication in ARPE-19 cells. *Rv3147* and *Rv3149* were uniquely regulated while *Rv3153* and *Rv3155* transcripts are also known to be upregulated in infected A549 cells (Ryndak et al., 2015), and *Rv3154* is upregulated in both A549 (Ryndak et al., 2015) and macrophage models (Rohde et al., 2012). Besides, the ATPase family has a critical role in the response of mycobacteria against the toxic substances in the phagosomal environment during active and latent infection (Novoa-Aponte and Soto Ospina, 2014). Two transcripts from the ATPase family (*CtpF: Rv1997* and *CtpG:1992c*) were found to be upregulated in the *in vitro* model of IOTB infection and *Rv1992c* transcript is reported to be upregulated in the macrophage model also (Rohde et al., 2012). Around 310 transcripts whose protein products constitute for cell wall component proteins, plasma membrane proteins, and cell wall biogenesis/organization were upregulated during the replication stage marking the growth of *M. tuberculosis* inside the host RPE cells (Supplementary Table 3). The MprAB system is also known to be involved in the activation of cellular processes associated with cell regrowth and in the regulation of cell wall component genes (He et al., 2006; Pang et al., 2007). However, the transcripts of MprAB was not found to be modulated in our study, which signifies that the change in these cell wall component protein is independent of the MprAB system and demands further analysis for regulation of these transcripts (Du et al., 2016). Certain transcripts whose proteins are involved in interaction with the host and performing symbiosis function during cellular processes were also found to be expressed (Supplementary Table 3). These results suggest that *M. tuberculosis* during its intracellular adaptation and growth is able to sustain the host-derived pressures by evolving its metabolic processes (Rohde et al., 2012).

Most genes upregulated from the cell wall and cell process and conserved hypothetical category are non-essential genes of *M. tuberculosis* during *in vitro* growth. It has been postulated that non-essential *M. tuberculosis* genes may have a potential role in *in vivo* survival and/or virulence (Jain et al., 2006). *Rv1230c* was among the top upregulated transcripts from the cell wall and cell processes functional category and is known to be involved in interacting with bacterial histone-like proteins which regulate many other genes involved in stress response and virulence factors (Gupta et al., 2014) and is also important for mycobacterial survival (Katsube et al., 2007). Presence of *Rv1230c* transcript (RNA) in 3 confirmed and 1 possible human IOTB cases (Table 3) suggests the presence of viable mycobacteria in vitreous samples of human IOTB cases. Interestingly, the protein encoded by Rv1230c has been reported as a potential drug target (Anand and Chandra, 2014). Two resuscitation-promoting factors (RpfC: Rv1884c and RpfE: Rv2450c) upregulated from the cell wall and cell processes functional category have earlier been reported to regulate the bacterial replication under stress conditions or modulate the host immune responses and contribute to mycobacterial pathogenesis (Rosser et al., 2017). Another upregulated gene *Rv2235* from the same category is predicted to be involved in the survival of mycobacteria inside the human macrophages (Miller and Shinnick, 2001). Among differentially regulated transcripts from PE and PPE families, the majority of the proteins encoded by the upregulated transcripts have an unknown function. However, it is reported that genes of this category occurs only in mycobacteria and may hold a key to command the immune pathogenesis (Brennan, 2017). Additionally, due to the presence of these proteins (antigens) on the mycobacterial cell surface, they elicit good antibodies and T-cell response (Copin et al., 2014; Brennan, 2017). PPE10 (Rv0442c) transcript whose protein product is involved in multiple cellular functions, such as in interaction with host and symbiosis functions (Supplementary Table 3), was upregulated in RPE cells. It is also found to be upregulated in infected A549 cells (Ryndak et al., 2015) and is involved in the maintenance of mycobacterial capsule layer with a significant role in virulence and immune modulation (Ates et al., 2016).

During mycobacterial growth, many biosynthesis pathways are active which are known to be involved in the synthesis of amino acids, fatty acids, and lipids as well as folate biosynthesis. Thus, the upregulation of transcripts whose protein products are involved in these metabolic activities (Supplementary Table 3) highlights the active metabolic physiological state of bacteria inside the host environment during replication. *Rv2672* encodes for a mycobacterial secretory protein with lipase and protease activities and hydrolyzes the host lipid for its nutrient requirements and helps in survival of *M. tuberculosis* inside lipid-rich foamy macrophages (Singh et al., 2017). Genes (*Rv0462*: *lpdC* and *Rv1005c*: *pabB*) of folate biosynthesis are predicted to be essential for mycobacterial survival (Griffin et al., 2011) and enzymes of folate metabolism are targets of potent antitubercular agents (Minato et al., 2015). *Rv0462* transcript is also known to be upregulated in *M. tuberculosis* infected A549 cells (Ryndak et al., 2015) and is involved in biosynthesis, energy, cellular process, and cell exterior pathways (Supplementary Table 3). Rv0462 contributes to induction of host innate and adaptive immunity (Heo et al., 2011) and plays a direct role in restricting the phagosome maturation (Philips, 2008). In parallel, the upregulation of many transcripts (*Rv2238c, Rv2874, Rv0462, Rv1676*, and *Rv3673c*) whose protein products have a role in oxidant detoxifications suggests the removal of toxic byproducts from fatty acid metabolism or another metabolic pathway (Supplementary Table 2) generated during active mycobacterial growth. It was observed that *Rv2238c, Rv1676*, and *Rv3673c* were uniquely upregulated in the *in vitro* model of IOTB while *Rv2874* is also known to be upregulated in the macrophage model (Rohde et al., 2012). Overall, 25 *M. tuberculosis* genes were upregulated from the functional category of virulence and detoxification (Figure 7D). Among these genes, four genes (*Rv1971, Rv0169, Rv0592*, and *Rv1967*) belong to mammalian cell entry (mce) family protein that plays a crucial role in invasion and virulence of *M. tuberculosis* (Zhang and Xie, 2011). Recently, *Rv1971* from this family was also reported to be upregulated in sputum samples of pulmonary TB patients (Sharma et al., 2017) and bioinformatically observed to be a highly antigenic protein involved in immune responses (Zhang et al., 2009). Interestingly, this gene was also detected in human IOTB samples (Table 3). Another mce gene *Rv0169* has been shown to be involved in the entry and survival of *M. tuberculosis* inside the nonphagocytic HeLa cells (Arruda et al., 1993; Saini et al., 2008), in cytoskeletal rearrangements (Chitale et al., 2001), and is also predicted to play a role in nutrient deficient environment inside the host cells (Pandey and Sassetti, 2008; Saini et al., 2008). Besides, the protein encoded by *Rv0169* is located on mycobacterial cell wall with its cell entry epitope flank on the surface (Das et al., 2003; Mitra et al., 2005) suggesting its role in host-pathogen interactions (Shimono et al., 2003). *Rv1755c* upregulated during intracellular replication of mycobacteria has predicted protein product with virulence function and is involved in the pathogenesis of *M. tuberculosis* for intracellular survival by altering the cell signaling events or directly causing cytotoxicity to host cells (Castro-Garza et al., 2016). Altogether, the upregulation of these transcripts controlling the various metabolic states of mycobacteria reflects the adaptation of *M. tuberculosis* to host immunity and environmental stresses in different conditions.

Besides, most of the significantly downregulated (through hypergeometric statistical evaluation with *p* < 0.05, Figure 7D) genes were from the functional categories of conserved hypothetical (135 genes), cell wall and cell process (133 genes), intermediary metabolism and respiration (133 genes), and lipid metabolism (51 genes), an indicator of decrease in metabolic state of *M. tuberculosis* soon after infection and internalization in host cells, which has been attributed to precursor of latency (Jain et al., 2006). Thus, analysis of the transcriptional profile of *M. tuberculosis* in RPE cells demonstrates the modulation of several transcripts whose protein products have earlier been reported to have a role in intracellular adaptation, survival, and multiplication in different *in vitro* experimental models of professional and non-professional phagocytic cells. These results not only favor the role of RPE cells as potential host cells for the survival of mycobacteria in the ocular environment but also suggest the role of the identified transcripts as potential targets for anti-tuberculous drugs and could also be exploited in IOTB detection. These findings indicate that *M. tuberculosis* growth is a programmed regulation of various metabolic pathways genes which helps in the survival and replication of bacteria inside RPE cells. Although the microarray technology used in the present study lead to the identification of a large number of differentially regulated *M. tuberculosis* transcripts in *in vitro* model of IOTB, it may not have detected all the differentially regulated transcripts as pre-designed complement probes against each gene (of *M. tuberculosis* genome) were used. The advancement in transcriptomic studies with technologies like RNA sequencing may add on to the current study.

In conclusion, this study demonstrates the invasion and replication of virulent strain of *M. tuberculosis* (H37Rv) in RPE cells, and the mycobacterial transcripts identified in the intracellular environment of RPE cells correspond to the pathways which have a crucial role in invasion, host adaptation, replication, and survival. As many of these transcript products are regulators of important metabolic pathways during *M. tuberculosis* growth and survival inside RPE cells, the results of this study may be important for unraveling the many unknown facets of IOTB pathogenesis.

## Author contributions

SA, IV, and AG conceptualized and designed the study. SA performed the experiments. SA and IV carried out data analysis, manuscript writing and editing. VG, RB and NS helped with studying human samples. US supported the TEM studies. SL arranged for resources and the training of SA for this study under the NIH funded Fogarty International Centre, USA (#D43 TW009588). AG and IV arranged for funding and resources.

### Conflict of interest statement

The authors declare that the research was conducted in the absence of any commercial or financial relationships that could be construed as a potential conflict of interest.

## Supplementary material

Supplementary Table 1 *Mycobacterium tuberculosis* genes specific primers used for a real time-polymerase chain reaction.

Supplementary Table 2 Transcriptional signatures of *M. tuberculosis* in an *in vitro* model of IOTB. *M. tuberculosis* transcripts differentially regulated in each functional category in an *in vitro* model of IOTB at 3-day post infection.

Supplementary Table 3 *Mycobacterium tuberculosis* proteins of biosynthesis, central dogma, energy, cellular process, and cell exterior pathways whose transcripts were upregulated (> ±1.15 Log2 FC) during intracellular multiplication of bacteria at 3-days post-infection in *in vitro* model of intraocular tuberculosis.

Supplementary Figure 1 Hierarchical clustering and principal components analysis of microarray data. **(A)** The hierarchical clustering of all the samples (infected and control) shows the replicability of the data obtained from each set. Six infected sets (3 biological and 3 technical replicate), isolated from infected ARPE-19 cells at 3-dpi and 2 control sets (1 biological and 1 technical) were evaluated for intracellular *M. tuberculosis* transcriptional signatures. **(B)** Principal components analysis of each sample (infected and control) was analyzed for correlation coefficient. The correlation coefficient data demonstrated the correlation between each respective set. The control sample (H37Rv RNA) was similar to its technical set, while all the infected sets (intracellular H37Rv RNA) showed similar correlation and thus were treated as replicates. **(C)** Values of co-relation coefficient obtained for each identifier (control and infected sample set). Dpi, days post infection. SG14484452_252632310045_1_1(Experiment-1), SG14484452_252632310045_1_2(Experiment-2), SG14484452_252632310045_1_3(Experiment-3), SG14484452_252632310045_1_4(Control-1), SG14484452_252632310045_2_1(technical repeat-1), SG14484452_252632310045_2_2(technical repeat-2), SG14484452_252632310045_2_3(technical repeat-3), and SG14484452_252632310045_2_4 (control technical repeat-1).
