# Supplementary material for: Transcriptional Profile of Mycobacterium tuberculosis in an in vitro Model of Intraocular Tuberculosis
Source: Front Cell Infect Microbiol. 2018 Oct 2;8:330. doi: 10.3389/fcimb.2018.00330 (PMC6175983; doi:10.3389/fcimb.2018.00330)
Supplement: Supplementary Table 1 — Mycobacterium tuberculosis genes specific primers used for a real time-polymerase chain reaction. [file Table_1.DOCX]

***Supplementary Table 1. Mycobacterium tuberculosis* gene specific primers used for real time-polymerase chain reaction**

| Gene | Forward primer 5'-3' | Reverse primer 5'-3' |
| --- | --- | --- |
| *Rv1230c* | CGACGCTGCTTTGGATGATG | GCGTCAGTTGGAACTTGCTG |
| *Rv1971* | CCAACCTCAACACCAAGCAG | GTTAAACGGCGAATCCTGGG |
| *Rv3872* | TTCACATCGGAGGGCATC | TCGTCGATTTGCGAATAGGT |
| *Rv2674* | AAGCTAGAACTGTCCGACGA | GAATTTCTCGGTGCTGCGG |
| *Rv2623* | ACGCACTCAAGGTGGTTGAA | ATCAGCACTGCGTCTTTGGA |
| *Rv3876* | CCGAAGGTGAAGAAGGTGAA | GGTCCAGCTCGTACTTCTCG |
| *Rv1965* | GGTCGCTCTGATGATCATGC | TAGGTCGCCAGGTGAAAGTT |
| *Rv3875* | AAGCGCAATCCAGGGAAATG | CGAACATCCCAGTGACGTTG |
| *Rv16s* | GTGGCGAACGGGTGAGTAAC | ATGCATCCCGTGGTCCTATC |
