# Supplementary figures and images for: Transcriptional Profile of Mycobacterium tuberculosis in an in vitro Model of Intraocular Tuberculosis
Source: Front Cell Infect Microbiol. 2018 Oct 2;8:330. doi: 10.3389/fcimb.2018.00330 (PMC6175983; doi:10.3389/fcimb.2018.00330)

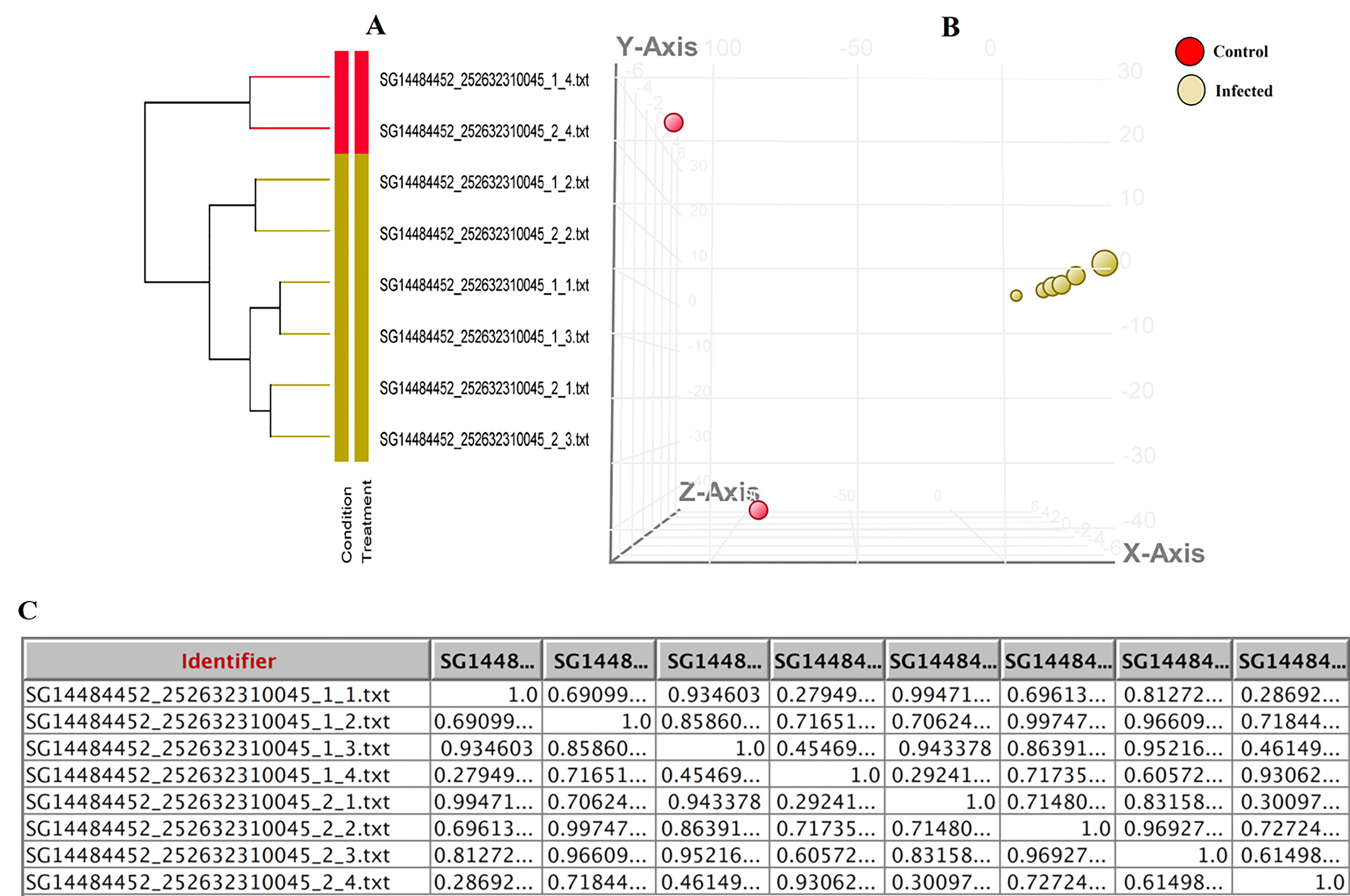

Supplement: Supplementary Figure 1 — Hierarchical clustering and principal components analysis of microarray data. (A) The hierarchical clustering of all the samples (infected and control) shows the replicability of the data obtained from each set. Six infected sets (3 biological and 3 technical replicate), isolated from infected ARPE-19 cells at 3-dpi and 2 control sets (1 biological and 1 technical) were evaluated for intracellular M. tuberculosis transcriptional signatures. (B) Principal components analysis of each sample (infected and control) was analyzed for correlation coefficient. The correlation coefficient data demonstrated the correlation between each respective set. The control sample (H37Rv RNA) was similar to its technical set, while all the infected sets (intracellular H37Rv RNA) showed similar correlation and thus were treated as replicates. (C) Values of co-relation coefficient obtained for each identifier (control and infected sample set). Dpi, days post infection. SG14484452_252632310045_1_1(Experiment-1), SG14484452_252632310045_1_2(Experiment-2), SG14484452_252632310045_1_3(Experiment-3), SG14484452_252632310045_1_4(Control-1), SG14484452_252632310045_2_1(technical repeat-1), SG14484452_252632310045_2_2(technical repeat-2), SG14484452_252632310045_2_3(technical repeat-3), and SG14484452_252632310045_2_4 (control technical repeat-1). [file Image_1.TIF]
